# Long-term outcomes of microsurgery and stereotactic radiosurgery as the first-line treatment for arteriovenous malformations: a propensity score-matched analysis using nationwide multicenter prospective registry data

**DOI:** 10.1097/JS9.0000000000000751

**Published:** 2023-09-14

**Authors:** Heze Han, Dezhi Gao, Li Ma, Ruinan Li, Zhipeng Li, Haibin Zhang, Kexin Yuan, Ke Wang, Yukun Zhang, Yang Zhao, Weitao Jin, Hengwei Jin, Xiangyu Meng, Debin Yan, Runting Li, Fa Lin, Qiang Hao, Hao Wang, Xun Ye, Shuai Kang, Jun Pu, Zhiyong Shi, Xiaofeng Chao, Zhengfeng Lin, Junlin Lu, Youxiang Li, Yuanli Zhao, Shibin Sun, Yu Chen, Xiaolin Chen, Shuo Wang

**Affiliations:** a Department of Neurosurgery; b Department of Gamma-Knife Center; c Department of Interventional Neuroradiology, Beijing Tiantan Hospital, Capital Medical University; d Department of Neurosurgery, Peking University International Hospital, Peking University; e China National Clinical Research Center for Neurological Diseases, Beijing; f Department of Neurosurgery, The First Hospital of Hebei Medical University, Hebei Medical University, Shijiazhuang; g Department of Neurosurgery, Shanxi Provincial People’s Hospital, Shanxi; h First Department of Neurosurgery, The Second Affiliated Hospital of Kunming Medical University, Kunming; i Department of Neurosurgery, Nanjing Drum Tower Hospital, Affiliated to Nanjing University, Nanjing, Jiangsu; j Department of Neurosurgery, The Second Affiliated Hospital of Xuzhou Medical University, Jiangsu; k Department of Neurosurgery, The First People’s Hospital of Qinzhou, Guangxi; l Department of Neurosurgery, West China Hospital, Sichuan University, Chengdu, Sichuan, People’s Republic of China

**Keywords:** arteriovenous malformation, hemorrhagic stroke, microsurgery, stereotactic radiosurgery, therapeutic strategy

## Abstract

**Background:**

This study aimed to compare the risk and benefit profile of microsurgery (MS) and stereotactic radiosurgery (SRS) as the first-line treatment for unruptured and ruptured arteriovenous malformations (AVMs).

**Materials and Methods:**

The authors included AVMs underwent MS or SRS as the first-line treatment from a nationwide prospective multicenter registry in mainland China. The authors used propensity score-matched methods to balance baseline characteristics between the MS and SRS groups. The primary outcomes were long-term hemorrhagic stroke or death, and the secondary outcomes were long-term obliteration and neurological outcomes. Subgroup analyses and sensitivity analyses with different study designs were performed to confirm the stability of our findings.

**Results:**

Of the 4286 consecutive AVMs in the registry from August 2011 to December 2021; 1604 patients were eligible. After matching, 244 unruptured and 442 ruptured AVMs remained for the final analysis. The mean follow-up duration was 7.0 years in the unruptured group and 6.1 years in the ruptured group. In the comparison of primary outcomes, SRS was associated with a higher risk of hemorrhagic stroke or death both in the unruptured and ruptured AVMs (unruptured: hazard ratio 4.06, 95% CI: 1.15–14.41; ruptured: hazard ratio 4.19, 95% CI: 1.58–11.15). In terms of the secondary outcomes, SRS was also observed to have a significant disadvantage in long-term obliteration [unruptured: odds ratio (OR) 0.01, 95% CI: 0.00–0.04; ruptured: OR 0.09, 95% CI: 0.05–0.15]. However, it should be noted that SRS may have advantages in preventing neurofunctional decline (unruptured: OR 0.56, 95% CI: 0.27–1.14; ruptured: OR 0.41, 95% CI: 0.23–0.76). The results of subgroup analyses and sensitivity analyses were consistent in trend but with slightly varied powers.

**Conclusions:**

This clinical practice-based real-world study comprehensively compared MS and SRS for AVMs with long-term outcomes. MS is more effective in preventing future hemorrhage or death and achieving obliteration, while the risk of neurofunctional decline should not be ignored.

## Introduction

Highlights

- Microsurgery is preferable in preventing future hemorrhage or death and achieving obliteration for arteriovenous malformations.
- Stereotactic radiosurgery may have advantages in preventing neurofunctional decline.
- Treatment strategies should be recommended for specific arteriovenous malformations with a comprehensive assessment.

Brain arteriovenous malformations (AVMs) are rare vascular lesions with abnormal connections between arteries and veins that bypass the capillary network^1^. Hemorrhage is recognized as the primary adverse event that could results in devastating complications if the lesion is left untreated^2^. Currently, the interventional management of AVMs include microsurgery (MS), stereotactic radiosurgery (SRS), and endovascular embolization^3^. Despite the advances in embolization in recent years, MS and SRS remain the first-line recommendation for most AVMs^4^.

The limitation of previous studies focusing on the comparison between the MS and SRS outcomes mainly derived from incomparable baselines and imbalanced follow-up duration^5^. Generally, MS was suggested for superficial, noneloquent AVMs, and SRS for the opposite ones^4,6,7^. Although many studies with matched data were conducted, confounders such as hemorrhage history and preoperative embolization were not well-balanced, and stratified analyses on beneficial profiles of the specific type of AVMs were unable to be performed due to limited sample sizes^4,8^. In addition, the trade-off between hemorrhage risk and neurological outcomes has long been debated in comparing the effect of MS and SRS^4,8–17^. MS was recommended for acute complete obliteration with a lower subsequent bleeding risk, and SRS for a minimally invasive operation with a lower neurological decline risk. Differences in prognostic assessment criteria often lead to inconsistent conclusions in previous studies^4,14^.

Given the unsolved confusion from previous studies, larger-scale studies with rigorous study designs are needed to confirm and expand upon these initial observations and provide more comprehensive insights into the choice of therapeutic strategy. In this clinical practice-based real-world study from a nationwide prospective multicenter registry, we compared the long-term risks of hemorrhagic stroke or death, obliteration rate, and neurofunctional outcomes between AVMs who received MS and SRS. We further analyzed AVMs categorized by different characteristics to provide references and indications for clinical practice.

## Methods

### Data sources and study design

This study was a retrospective analysis of a prospectively maintained nationwide multicenter registry, MATCH study (the registry of multimodality treatment for brain AVMs in mainland China, NCT 04572568) from August 2011 to December 2021. The objective of this registry was to analyze the natural progression of AVMs in the Asian population and determine the most effective individualized treatment strategy for patients with AVMs. Previous protocol and publications have proved the validity of the registry^18–20^. A detailed procedure of data quality management was shown in Supplementary Method 1 (Supplemental Digital Content 1, http://links.lww.com/JS9/A993).

In this study, patients with AVMs undergoing MS and SRS as their first treatment were eligible for inclusion. The exclusion criteria were as follows: patients missing critical clinical baseline data, pretreatment imaging, and available post-treatment radiological and clinical outcomes data; patients undergoing endovascular embolization or hybrid operation (MS with one-staged preoperative embolization or angiography) as their first treatment; patients undergoing emergency palliative partial resection with expanding hematoma or deteriorating consciousness, as in these situations MS but not SRS were mandatory, and the inclusion of them would violate the positivity assumption of the propensity score methods^21^. This study obtained the approval of the institutional ethics committee (IRB approval number: KY 2020-003-01) in compliance with the 1964 Helsinki Declaration. All patients who enrolled in the registry gave their written informed consent at admission. This study followed the strengthening the reporting of cohort, cross-sectional, and case–control studies in surgery (STROCSS) guidelines for observational cohort studies^22^ (Supplemental Digital Content 2, http://links.lww.com/JS9/A994).

### Cohort definition

We divided the AVMs into two groups according to their first therapeutic strategy, MS and SRS, to compare the risk and benefit profiles of these two interventions. MS was performed with the goal of complete resection and cure, and the cases that had emergency palliative partial resection were excluded. For patients undergoing MS, routine preoperative catheter angiography and MRI were required for surgical planning. The resections with standard microsurgical techniques were carried out by neurosurgeons with more than 15 years of expertise in the cerebrovascular field. Postoperative hemorrhage was evaluated via head CT within 12 h of MS. In cases selected for SRS, all procedures were performed using the Gamma Knife, with stereotactic planning neuroimaging results imported into the Leksell Gamma-Plan workstation (Elekta AB, Elekta Company). The specific model of Gamma Knife utilized was determined by its availability at the respective institution participating in the registry. The procedures of SRS were performed in a similar manner across all centers. In brief, a stereotactic frame was attached to the patient’s head prior to the procedure for positioning. High-resolution and thin-slice (slice thickness 1–2 mm) MRI scans were obtained for the planning. The pre-SRS AVM MRI protocol consisted of at least three sequences: T1-weighted imaging, contrast-enhanced T1-weighted imaging, and T2-weighted imaging. The SRS treatment plan was based on the agreement of two senior neuroradiologists with experience. The radiation target was delineated using these sequences on 3D stereotactic MRI, and the dose planning was made according to the location and volume of AVMs. Based on hemorrhage presentation before the first treatment, we identified two different subcohorts which comprised unruptured and ruptured AVMs.

### Baseline characteristics

The study collected data on demographic variables (age and sex), clinical manifestations [seizure, neurological deficiency, and modified Rankin Scale (mRS) at admission], and radiological characteristics of AVMs (location, size, and angiographic features of feeding arteries, the nidus, and drainage veins). The radiological characteristics were defined according to the reporting terminology guidelines^23^, and assessed by digital subtraction angiography (DSA) and MRI. The angio-architectural parameters were verified by neurosurgery residents who received training from qualified senior neuroradiologists using pre-interventional imaging.

### Outcomes and follow-up

The primary outcome was the composite event of hemorrhagic stroke or death during follow-up. Hemorrhagic stroke referred to the clinically symptomatic event (any new focal neurological deficit, seizure, or new-onset severe headache) validated by imaging confirmation (intracranial hematoma or subarachnoid hemorrhage that could be associated with AVM on computed tomography or MRI). Hemorrhage at the postoperative acute phase (within 2 weeks immediately after the procedures) was not included in the primary outcome as we aimed to investigate the long-term efficacy of the treatment. For the death outcome, we only considered those cases that were related to AVMs. The secondary outcomes were obliteration rate and neurofunctional outcomes at follow-up. AVM obliteration was defined as a lack of abnormal flow voids on MRI or an absence of anomalous arteriovenous shunting on DSA. Neurofunctional outcomes were assessed via the mRS system. The disabling neurological deficit was defined as an mRS greater than 2 at the final follow-up. Neurofunctional decline was defined as a worsening mRS at follow-up compared with admission.

Clinical outcomes were assessed by phone interviews or record review by experienced clinical research coordinators at 3 months, annually (1, 2, and 3 years), and every 5 years after the treatment. To minimize follow-up bias, we employed a variety of strategies aimed at retaining participants and maximizing follow-up completion, including flexible follow-up methods and statistical analysis for non-responders (Supplementary Method 2, Supplemental Digital Content 1, http://links.lww.com/JS9/A993). The inception point of the follow-up was the date of first treatment, and the endpoint was the date of hemorrhagic stroke/death, treatment switching to other modalities, or the last follow-up, whichever came first. Radiological outcome results were assessed by two experienced neuroradiologists.

### Controlling for confounding

To reduce the effects of potential confounding bias, we used propensity scores to control for pretreatment imbalances on baseline characteristics. These factors that could potentially affect both the selection of particular therapeutic strategies and outcomes were specifically referred to as ʻconfounding by indicationʼ^24^. Propensity score matching (PSM) methods were recommended to address this issue in clinical research^25^. All available baseline characteristics, including demographic factors, clinical presentation at admission, morphological features, and angio-architectural parameters, were matched between the MS and the SRS group separately in the unruptured and the ruptured subcohorts. In this study, we used the nearest-neighbor method with the caliper radius of 0.2 without replacement to do 1:1 patient matching. Covariate balance was assessed with standardized mean differences (SMD) with an acceptable balance set at values less than 0.1. Even with PSM, residual confounding from unmeasured factors could remain. Thus, we used *E*-values to assess how strong such factors would have to be to nullify the observed association^26^.

### Statistical analyses

All analyses were implemented using R software (version 4.2.2). Statistical significance was set at two-sided *P*<0.05. For datasets before PSM, we assumed missingness at random. These missing values were imputed using the missForest package for R software, a nonparametric approach for mixed-type data. Baseline characteristics of the MS and the SRS groups before and after PSM were compared. Continuous variables were reported as mean±SD or median [interquartile ranges (IQR)] for normal and non-normal distribution data. The proportion (*n*%) of each categorical variable was also recorded. All the subsequent analyses were conducted using post-PSM cohorts, except for the last sensitivity analysis with stabilized inverse probability of treatment weighting (sIPTW). Kaplan–Meier curves were plotted to visualize the cumulative incidence of primary outcomes and log-rank tests were used to compare hazard rates between treatment strategies. We calculate the attributable risks (ARs) for all outcomes after PSM. For the primary outcomes, we tabulated the numbers of events, incidence rates, and ARs referred to rate differences per 100 person-years. Hazard ratios (HRs) with 95% CIs were also estimated using Cox proportional hazard models. For secondary outcomes, ARs were recognized as risk differences, and odds ratios (ORs) were calculated using the logistic regression model.

To investigate whether specific AVMs could benefit from MS or SRS after weighting the hemorrhage risk and neurofunctional outcomes, prespecified subgroup analyses were conducted according to the age at diagnosis (<18 years or ≥18 years), eloquent regions (yes or no), Spetzler–Martin (S–M) grade (I–II, III, or IV–V), and supplemented S–M grade (≤VI or >VI). Comparison of treatment efficacy categorized by detailed S–M grade and supplemented S–M grade were also calculated using ARs. We also inspected the robustness of the main findings through several sensitivity analyses. First, an analysis excluding patients switching to other treatment modalities after their first MS or SRS procedure was conducted. Second, to investigate the efficacy of single-session procedures, patients undergoing multiple MS or SRS treatment were excluded in this sensitivity analysis. Third, to reduce the effect of the latency period in SRS patients, we included SRS patients only with a follow-up duration of more than three years. Fourth, to examine the reliability of propensity score methods, we applied the sIPTW, another approach recommended for confounder controlling, to compare the two treatment strategies. The PSM methods were used in the first three analyses.

## Results

### Study population and baseline characteristics

**Figure 1:**
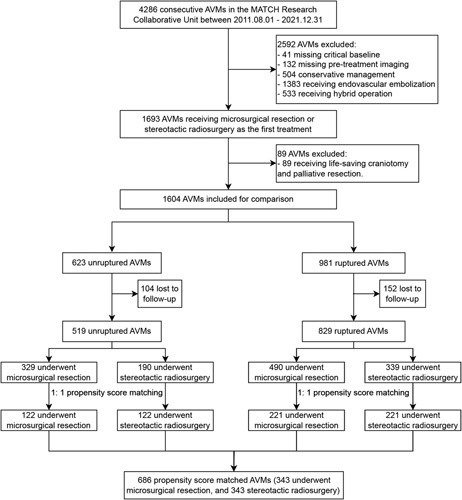
Flowchart of patient selection. AVM, arteriovenous malformation.

A total of 4286 patients diagnosed with AVMs were enrolled in the MATCH registry between August 2011 and December 2021. After careful screening, we included 1348 patients with complete clinical follow-up for final analyses (519 unruptured and 829 ruptured AVMs). The distribution of imputed missing data was shown in Supplementary Table 1 (Supplemental Digital Content 1, http://links.lww.com/JS9/A993). The detailed baseline comparisons between the included patients and those lost to follow-up were listed in Supplementary Table 2 (Supplemental Digital Content 1, http://links.lww.com/JS9/A993), with no significant differences between them. And the breakdown of patients lost to follow-up grouped by MS and SRS treatment was shown in Supplementary Table 3 (Supplemental Digital Content 1, http://links.lww.com/JS9/A993). PSM was applied separately in the unruptured and the ruptured subcohorts, which resulted in 244 unruptured (122 per intervention group) and 442 ruptured AVMs (221 per intervention group). And the overall post-PSM population was formed by combining the two post-PSM subcohorts (686 cases). Figure 1 shows the details of how patients were selected.

Before PSM, patients were more likely to choose SRS if the AVMs were in infratentorial or eloquent regions, with perforating artery supplies or with deep vein drainage. After PSM, all these recorded baseline characteristics were comparable in all the three cohorts, as shown in Supplementary Table 4 (Supplemental Digital Content 1, http://links.lww.com/JS9/A993). And the balance test plotted in the Supplementary Figure 1 (Supplemental Digital Content 1, http://links.lww.com/JS9/A993) also indicated that an acceptable balance was achieved between the two treatments. Seizures are more frequent in unruptured AVMs (MS vs SRS: 46.7 vs 41.8%) comparing with ruptured AVMs (MS vs SRS: 14.9 vs 15.4%). Most AVMs were of S-M I–III grades (MS vs SRS: 89.3 vs 90.2% in the unruptured AVMs; 89.6 vs 88.2% in the ruptured AVMs; 89.5 vs 88.9% in the overall cohort). And half of the AVMs were seated in eloquent regions (MS vs SRS: 49.2 vs 50.0% in the unruptured AVMs; 50.2 vs 53.8% in the ruptured AVMs; 49.9 vs 52.5% in the overall cohort). The mean (SD) follow-up duration of the overall cohort was 6.4 (4.0) years [unruptured: 7.0 (4.4) years; ruptured: 6.1 (3.7) years]. The follow-up duration of different treatments was similar both in the unruptured and ruptured groups. Details on follow-up and censoring reasons are reported in Supplementary Table 5 (Supplemental Digital Content 1, http://links.lww.com/JS9/A993).

### Primary outcomes

After PSM, the incidence rates per 100 person-years for hemorrhagic stroke or death were overall 0.36 vs 1.65 in MS vs SRS, respectively, showing among patients undergoing SRS a four times increased risk compared with MS [HR, 4.28 (95% CI: 1.97–9.30)], or one more event in 100 person-years [AR, 1.29 (95% CI: 0.70–1.89) per 100 person-years]. The results across subcohorts were consistent with the overall findings [unruptured AVMs: HR, 4.06 (95% CI: 1.15–14.41); AR, 1.17 (95% CI: 0.25–2.09) per 100 person-years; ruptured AVMs: HR, 4.19 (95% CI: 1.58–11.15); AR, 1.37 (95% CI: 0.59–2.15) per 100 person-years] (Table 1). The association between SRS and increased AVM-related death was not of statistical significance due to limited event counts. And the SRS treatment saw more symptomatic hemorrhagic stroke during follow-up [overall population: HR, 4.90 (95% CI: 2.16–11.12); AR, 1.34 (95% CI: 0.75–1.92) per 100 person-years; unruptured AVMs: HR, 6.11 (95% CI: 1.37–27.32); AR, 1.29 (95% CI: 0.40–2.18) per 100 person-years; ruptured AVMs: HR, 4.19 (95% CI: 1.58–11.15); AR, 1.37 (95% CI: 0.59–2.15) per 100 person-years]. The *E*-values of all the significant results indicated that unmeasured confounders required a strong association with treatment and the primary outcome to explain away the observed association.

**Table 1:** Outcomes of different therapeutic strategies after propensity score matching.

	MS (%)	SRS (%)	Attributable risk^a^ (95% CI)	*P*	HR (95% CI)/ OR (95% CI)^a^	*P*	*E*-value (95% CI)^b^
Unruptured AVMs	122	122					
Primary outcomes							
Hemorrhage stroke or death	3 (0.36)	13 (1.52)	1.17 (0.25–2.09)	0.013	4.06 (1.15–14.41)	0.030	7.58 (6.01–9.15)
symptomatic hemorrhagic stroke	2 (0.24)	13 (1.52)	1.29 (0.40–2.18)	0.005	6.11 (1.37–27.32)	0.018	11.70 (9.62–13.78)
AVM-related death	1 (0.12)	4 (0.47)	0.35 (−0.16–0.86)	0.183	4.17 (0.47–37.31)	0.202	–
Secondary outcomes							
Obliteration	119 (97.54) *n*=122	26 (29.21) *n*=89	−68.33 (−78.17–−58.49)	<0.001	0.01 (0.00–0.04)	<0.001	199.50 (150.01–248.99)
Disability	7 (5.74)	6 (4.92)	−0.82 (−6.45–4.81)	0.776	0.85 (0.28–2.61)	0.776	–
Neurofunctional decline	23 (18.85)	14 (11.48)	−7.38 (−16.33–1.58)	0.106	0.56 (0.27–1.14)	0.111	–
Ruptured AVMs	221	221					
Primary outcomes							
Hemorrhage stroke or death	5 (0.36)	23 (1.73)	1.37 (0.59–2.15)	0.001	4.19 (1.58–11.15)	0.004	7.85 (5.31–10.39)
symptomatic hemorrhagic stroke	5 (0.36)	23 (1.73)	1.37 (0.59–2.15)	0.001	4.19 (1.58–11.15)	0.004	7.85 (5.31–10.39)
AVM-related death	0 (0.00)	1 (0.08)	0.08 (−0.07–0.22)	0.317	–	–	
Secondary outcomes							
Obliteration	201 (90.95) *n*=221	84 (47.19) *n*=178	−43.76 (−52.01–−35.51)	<0.001	0.09 (0.05–0.15)	<0.001	21.71 (8.90–34.52)
Disability	19 (8.60)	13 (5.88)	−2.71 (−7.54–2.11)	0.270	0.66 (0.32–1.38)	0.273	–
Neurofunctional decline	37 (16.74)	17 (7.69)	−9.05 (−15.10–−3.00)	0.003	0.41 (0.23–0.76)	0.005	4.31 (2.35–6.27)
Overall population	343	343					
Primary outcomes							
Hemorrhage stroke or death	8 (0.36)	36 (1.65)	1.29 (0.70–1.89)	<0.001	4.28 (1.97–9.30)	<0.001	8.03 (4.68–11.38)
Symptomatic hemorrhagic stroke	7 (0.32)	36 (1.65)	1.34 (0.75–1.92)	<0.001	4.90 (2.16–11.12)	<0.001	9.27 (5.53–13.01)
AVM-related death	1 (0.05)	5 (0.23)	0.18 (−0.04–0.40)	0.100	5.13 (0.60–43.89)	0.136	–
Secondary outcomes							
Obliteration	320 (93.29) *n*=343	110 (41.20) *n*=266	−52.10 (−58.52–−45.63)	<0.001	0.05 (0.03–0.08)	<0.001	39.49 (15.00–63.98)
Disability	26 (7.58)	19 (5.54)	−2.04 (−5.74–1.66)	0.280	0.71 (0.39–1.32)	0.282	–
Neurofunctional decline	60 (17.49)	31 (9.04)	−8.45 (−13.49–−3.42)	0.001	0.47 (0.30–0.74)	0.001	3.68 (1.56–5.80)

a The results were calculated with the MS group as the reference. The metrics of the primary outcomes were expressed as rate per 100 patient years and hazard ratios, and the secondary outcomes were expressed as proportion and odds ratios.

b *E*-values were calculated for HRs or ORs with statistical significance.

AVM, arteriovenous malformation; HR, hazard ratio; MS, microsurgery; OR, odds ratios; SRS, stereotactic radiosurgery.

**Figure 2:**
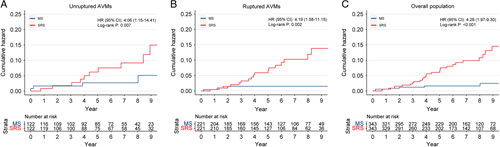
Cumulative incidence for hemorrhagic stroke or death by therapeutic strategies in unruptured (A), ruptured (B), and overall populations (C). AVM, arteriovenous malformation; HR, hazard ratio; MS, microsurgery; SRS, stereotactic radiosurgery.

Kaplan–Meier curves comparing the cumulative incidence of hemorrhagic stroke or death between MS and SRS treatment were consistent with these results and across subcohorts (Fig. 2). Interestingly, we found that the postoperative hemorrhage risk of SRS increased proportionally over time, while the hemorrhage risk of MS reached a plateau after two years postoperatively, which makes MS superior to SRS in controlling the long-term hemorrhage risk.

### Secondary outcomes

Among these patients with clinical follow-up, radiological follow-up was missing in 76 (11.08%) patients, all of whom were in the SRS groups. Overall, the obliteration rates were significantly higher in patients receiving MS (MS vs SRS: 93.29 vs 41.20%). In the MS treatment, ruptured AVMs subcohorts represented a decreased obliteration rate compared with the unruptured group (ruptured vs unruptured AVMs: 90.95 vs 97.54%), while in the SRS treatment, the opposite phenomenon was observed (ruptured vs unruptured AVMs: 47.19 vs 29.21%) (Table 1).

For the disabling neurological deficit outcome, MS and SRS did not differ significantly in all comparisons after PSM [overall population: OR, 0.71 (95% CI: 0.39–1.32); unruptured AVMs: OR, 0.85 (95% CI: 0.28–2.61); ruptured AVMs: OR, 0.66 (95% CI: 0.32–1.38)]. However, SRS treatment was observed to reduce the risk of neurofunctional decline in the overall population [OR, 0.47 (95% CI: 0.30–0.74)] and in ruptured AVMs [OR, 0.41 (95% CI: 0.23–0.76)]. The effect of SRS on neurofunctional decline in unruptured AVMs was also protective, but insignificant with a wider CI [OR, 0.56 (95% CI: 0.27–1.14)].

### Subgroup analyses and sensitivity analyses

**Figure 3:**
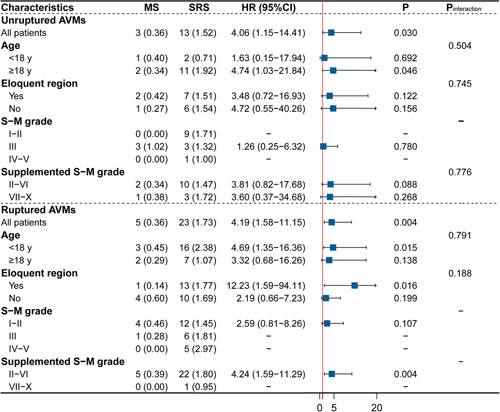
Subgroup analysis for primary outcomes. AVM, arteriovenous malformation; HR, hazard ratio; mRS, modified Rankin Scale; MS, microsurgery; S–M grade, Spetzler–Martin grade; SRS, stereotactic radiosurgery.

Results of the comparison of MS and SRS on hemorrhagic stroke or death were consistent across subgroups with no interaction effect (Fig. 3). For unruptured AVMs, all results but the adult subgroup were insignificant as the degree of uncertainty was higher and the point estimates were less precise due to the reduced statistical power. For ruptured AVMs, those eloquent-seated [HR, 12.23 (95% CI: 1.59–94.11)], with younger ages [HR, 4.69 (95% CI: 1.35–16.36)] and lower supplemented S–M grades [HR, 4.24 (95% CI: 1.59–11.29)] were more likely to benefit from MS in the prevention of subsequent hemorrhage or death. Supplementary Figure 2 (Supplemental Digital Content 1, http://links.lww.com/JS9/A993) shows the comparisons of neurological outcomes across subgroups. In the subgroup analysis of neurofunctional decline, an interaction effect was found between treatment strategies and S–M grades in the unruptured AVMs (*P* for interaction=0.017). Unruptured AVMs with a supplemented S–M grade higher than VI were less likely to suffer from neurofunctional decline if treated by SRS [OR, 0.20 (95% CI: 0.05–0.84)].

Supplementary Tables 6–8 (Supplemental Digital Content 1, http://links.lww.com/JS9/A993) provides the comparisons categorized by detailed S–M grade. The differences in both hemorrhagic stroke or death and neurofunctional outcomes in the unruptured AVMs all failed to reach statistical significance in all these detailed subgroups due to limited samples and events. However, in the ruptured AVMs, MS may be a better choice in S–M IV (S2E1V1 subtype) grade for significantly lower hemorrhage risk and similar neurological outcomes. On the contrary, in ruptured S–M III (S1E1V1 and S2E0V1subtype) grade, SRS was more recommended for significantly lower risks of worse neurological outcomes but similar hemorrhage risks.

The sensitivity analyses (Supplementary Table 9, Supplemental Digital Content 1, http://links.lww.com/JS9/A993) demonstrated the comparative effect of the treatments under different study designs. In both unruptured and ruptured subcohorts, MS was consistently observed to have stable advantages over SRS in preventing long-term bleeding or death, as well as long-term occlusion rates. Additionally, SRS was found to be superior in preventing neurofunctional decline under different study designs.

## Discussion

In this observational cohort study from a nationwide multicenter registry, we compared the long-term outcomes of MS and SRS as the initial treatment for unruptured and ruptured AVMs. This study found that MS had a lower risk of hemorrhagic stroke or death and a higher obliteration rate than SRS, while SRS had a lower risk of neurofunctional decline, no matter in the unruptured and ruptured AVMs. The results were stable across different subgroups and study designs. We also identified specific AVMs that could benefit from MS or SRS after balancing the hemorrhage risk and neurofunctional outcomes. These findings inform individualized treatment selection for patients with AVMs.

In a previous meta-analysis investigating the comparative effectiveness and safety of MS and SRS for AVMs, MS was recommended as a first-line consideration for higher obliteration rate, lower hemorrhage risk, and similar neurological outcomes^5^. However, most of the included studies used unmatched data, which meant that the choice of therapeutic strategies was carefully selected and their results may not be directly comparable due to confounders by treatment indication^13–17^. Only two studies used a matched design to compare the two treatment strategies^4,8^. Limited by the sample sizes, both studies failed to distinguish stand-alone treatments from those with preoperative embolization whose effect on the MS and SRS remains debatable^27–31^. The average follow-up durations in these two studies were also imbalanced with the MS group only observed for less than 1 year and the SRS group for 3–7 years. This, on the one hand, reflected the bias toward the use of SRS at their centers, and on the other hand, could potentially underestimate the hemorrhage risk and overestimate the post-treatment neurological deficit of MS over time. As such, in spite of the numerous literature reports on the pros and cons of MS and SRS, this multicenter large-sample matched study was conducted to enhance the level of evidence, and to investigate optimal treatment for specific AVMs.

The treatment goal of AVMs was to prevent hemorrhage by completely obliterating the nidus^32,33^. In Nerva *et al*.^17^ study, they did not distinguish the early postresection hemorrhage from long-term hemorrhage, and concluded that MS bears higher risks than SRS. Therefore, we set the hemorrhagic stroke or death at the follow-up period as the primary outcome to avoid bias brought by technical inadequacy^5^. In accordance with most previous studies, our results also demonstrated a beneficial effect of MS in preventing hemorrhage^5^. For unruptured AVMs, the protective effect of MS was statistically insignificant in some sensitivity analyses with a wider CI, but the HR remained favoring MS. This could result from limited events and sample sizes in the matched subcohorts. From the perspective of preventing rupture, MS would be recommended as a first-line consideration as the hemorrhage risk after SRS caused by residual AVMs or the latency period effect remains stable beyond the follow-up duration^34,35^. Previous researches have seen higher risk of stroke or death during the early postsurgery stage^14,36,37^. In this study, KM curves also represent a similar pattern with hemorrhagic events slightly more frequently happened in MS-treated AVM in the first 2 years after MS, but gradually disappeared thereafter.

The obliteration rates in this study were unsurprisingly higher in the MS-treated AVMs than in the SRS-treated ones across subcohorts and subgroups. The overall obliteration rate was in consistent with previous reports with MS groups more than 90% and SRS groups varying widely from 0 to 78%^3,5,38^. The rates were lower in our SRS cohort (overall, 41.20%; unruptured AVMs, 29.21%; ruptured AVMs, 47.19%) than other studies, which could be explained by relatively lower margin doses applied in our registry [median (IQR), Gy: overall, 17.0 (16.0, 18.0); unruptured AVMs, 16.0, (15.0, 17.0); ruptured AVMs, 17.0 (16.0, 18.0)]^39^. In the sensitivity analysis excluding SRS-treated patients with less than 3 years follow-up, the obliteration rates were seen to increase (overall, 45.74%; unruptured AVMs, 36.25%; ruptured AVMs, 51.05%).

Potential neurological impairment brought by the invasive procedures was the main reason that impede MS from being the first-line treatment for all AVMs, which was also in turn the advantage of SRS. However, the minimally invasive nature of SRS did not spare its risk of neurological deficit after the procedure. The deficits after MS and SRS typically happened in acute and delayed phases of the postoperative follow-up period, respectively^5^. As such, the variation in post-treatment neurological deficit rates between the two treatment modalities might lessen over time with the MS-treated patients undergoing neurological rehabilitation or the SRS-treated patients experiencing delayed complications^4^. Consequently, for neurosurgeons, the critical issue is to detect specific AVM subtypes that have the lowest risk of postoperative functional impairment. For example, in the subgroup analysis, our results suggest that unruptured AVMs with lower supplemented S–M grade (II–VI) would benefit more if treated by MS after the hemorrhage-neurological function trade-off, which was in line with Kim *et al*.^40^ recommendation. This study also provided more detailed comparisons in specific subtypes categorized by S–M grade and supplemented S–M grade, with most of the results being insignificant differences due to less statistical power. Despite that, the cautious interpretation could serve as a reference for doctors and patients.

This research has several strengths in study design. First, it is based on a large-sample size with AVMs from a prospective multicenter registry, and with balanced provider experience for both treatments, which increases the statistical power of the main results. Second, we excluded patients who had palliative resection in an emergency because of hematoma compression or progressive consciousness decline, reducing the impact of this incomparable situation on the outcome. Third, PSM was applied to control for potential confounding by indication, which reduces the bias due to nonrandomized treatment selection. And for the underlying unmeasured confounders, we calculated the *E*-value to assess how strong they would have to be to explain away the observed associations, which enhances the robustness and credibility of the results. Fourth, we conducted several sensitivity analyses with different study designs to investigate whether methodological variance could result in different comparative effects between the two treatment strategies.

However, our study still has some limitations. First, postoperative complications were not identified as outcomes in this study, as the types of complications varies differently in these two treatments, making the comparison difficult. For SRS, long-term complications like second primary cancer, radiation-induced change, and delayed cyst formation were also not evaluated. Future studies considering this important aspect could provide a more comprehensive view of the long-term risks associated with SRS. Second, the dose planning for SRS was not standardized across the participating centers, and was made by experienced neuroradiologists. This lack of standardization could potentially affect the outcomes, and future research could benefit from protocolized dose planning to enhance study quality. Third, although mRS was widely used to evaluate the neurofunctional outcome, it was not designed to assess AVM outcomes. Future studies with scales specialized for AVMs or considering outcomes beyond hemorrhage, neurofunctional status, or obliteration rate were encouraged. Fourth, there may be variability in the radiological outcomes due to lost-to-follow-up or different imaging modalities to assess AVM obliteration. However, previous studies also demonstrated the similar accuracy of MRI and DSA in evaluating nidal patency^41,42^. And follow-up compliance should also be considered in the AVM management and in future studies. Fifth, regardless of using methodological measures to reduce confounding in this observational study, the causal effects of MS and SRS on AVM outcomes should be confirmed by randomized controlled trials.

## Conclusions

In conclusion, our study showed that MS and SRS have different risk-benefit profiles for patients with AVMs. MS is more effective in preventing future hemorrhage or death and achieving obliteration, while neurofunctional decline should not be ignored. The subgroup analysis found consistent treatment effects with varied powers in specific AVMs, suggesting the need for individualized treatment planning based on a comprehensive assessment of AVMs.

## Ethical approval

This study was approved by Institutional Research Ethics Committee of Beijing Tiantan Hospital (IRB approval number: KY 2020-003-01) and conducted under the guidance of the Declaration of Helsinki.

## Consent

All patients who enrolled in the registry gave their written informed consent at admission.

## Sources of funding

This work was supported by the National Key Research and Development Program of China (Grant No. 2021YFC2501101 and 2020YFC2004701 to Xiaolin Chen), Natural Science Foundation of China (Grant No. 81771234 and 82071302 to Yuanli Zhao, and 82202244 to Yu Chen), and Top Talent Support Program for Medical Experts Team of Wuxi Health Committee (Grant No. 202109 to Shuo Wang). The study sponsors had no involvement in the collection, analysis and interpretation of data, in the writing of the manuscript; or in the decision to submit the manuscript for publication.

## Author contribution

H.H.: methodology, software, validation, formal analysis, investigation, visualization, data curation, and writing – original draft; D.G.: resources, methodology, validation, and investigation; L.M.: methodology, validation, and investigation; R.L. and Z.L.: resources and investigation; H.Z., K.Y., and K.W.: resources; Y.Z. and Y.Z.: resources, validation, and investigation; W.J.: validation and investigation; H.J. and X.M.: investigation; D.Y.: resources; R.L., J.P., Z.S., X.C., Z.L., J.L., and F.L.: resources and investigation; Q.H., H.W., X.Y., and S.K.: methodology; Y.Z.: conceptualization, supervision, funding acquisition; S.S.: conceptualization and supervision; Y.C.: conceptualization, methodology, formal analysis, investigation, writing – original draft; X.C.: conceptualization, supervision, funding acquisition; S.W.: conceptualization, funding acquisition, writing – review and editing, supervision, project administration. All authors confirm that they contributed to manuscript reviews and critical revision for important intellectual content, and read and approved the final draft for submission. All authors agree to be accountable for the content of this study.

## Conflicts of interest disclosure

There are no conflicts of interest.

## Research registration unique identifying number (UIN)

1. Name of the registry: Registry of multimodality treatment for brain AVMs in mainland China (MATCH).
2. Unique identifying number or registration ID: ClinicalTrials.gov register, NCT 04572568.
3. Hyperlink to your specific registration (must be publicly accessible and will be checked): https://www.clinicaltrials.gov/ct2/show/NCT04572568?cond=04572568&draw=2&rank=1.

## Guarantor

Shuo Wang, MD, Department of Neurosurgery, Beijing Tiantan Hospital, Capital Medical University, Beijing, People’s Republic of China. E-mail: captain9858@126.com.

## Data availability statement

All original data are available upon reasonable request to the corresponding authors.

## Provenance and peer review

This paper was not invited.

## Supplementary Material

SUPPLEMENTARY MATERIAL

## References

[1] Lawton MT Rutledge WC Kim H. Brain arteriovenous malformations. Nat Rev Dis Primers 2015;1:15008. 27188382 10.1038/nrdp.2015.8

[2] Solomon RA Connolly ES. Arteriovenous malformations of the brain. N Engl J Med 2017;376:1859–1866. 28489992 10.1056/NEJMra1607407

[3] van Beijnum J van der Worp HB Buis DR. Treatment of brain arteriovenous malformations: a systematic review and meta-analysis. JAMA 2011;306:2011–2019. 22068993 10.1001/jama.2011.1632

[4] Chen CJ Ding D Wang TR. Microsurgery versus stereotactic radiosurgery for brain arteriovenous malformations: a matched cohort study. Neurosurgery 2019;84:696–708. 29762746 10.1093/neuros/nyy174

[5] Sattari SA Shahbandi A Kim JE. Microsurgery Versus Stereotactic Radiosurgery for Treatment of Patients With Brain Arteriovenous Malformation: A Systematic Review and Meta-Analysis. *Neurosurgery*. Published online March 31, 2023. 10.1227/neu.0000000000002460 36999929

[6] Mascitelli JR Yoon S Cole TS. Does eloquence subtype influence outcome following arteriovenous malformation surgery? J Neurosurg 2018;131:876–883. 30497229 10.3171/2018.4.JNS18403 PMC6800816

[7] Schramm J Schaller K Esche J. Microsurgery for cerebral arteriovenous malformations: subgroup outcomes in a consecutive series of 288 cases. J Neurosurg 2017;126:1056–1063. 27285541 10.3171/2016.4.JNS153017

[8] Nataf F Schlienger M Bayram M. Microsurgery or radiosurgery for cerebral arteriovenous malformations? A study of two paired series. Neurosurgery 2007;61:39–49. 17621017 10.1227/01.neu.0000279722.60155.d3

[9] Steiner L Lindquist C Cail W. Microsurgery and radiosurgery in brain arteriovenous malformations. J Neurosurg 1993;79:647–652. 8410242 10.3171/jns.1993.79.5.0647

[10] Gross BA Duckworth EAM Getch CC. Challenging traditional beliefs: microsurgery for arteriovenous malformations of the basal ganglia and thalamus. Neurosurgery 2008;63:393–410. 18812951 10.1227/01.NEU.0000316424.47673.03

[11] Ding D Yen CP Xu Z. Radiosurgery for low-grade intracranial arteriovenous malformations. J Neurosurg 2014;121:457–467. 24605839 10.3171/2014.1.JNS131713

[12] Gami A Feghali J Rapaport S. Microsurgical resection versus stereotactic radiosurgery for low-grade intracranial arteriovenous malformations: A 27-year institutional experience. J Clin Neurosci 2021;94:209–215. 34863440 10.1016/j.jocn.2021.10.036

[13] Chen Y Yan D Li Z. Long-term outcomes of elderly brain arteriovenous malformations after different management modalities: a multicenter retrospective study. Front Aging Neurosci 2021;13:609588. 33679374 10.3389/fnagi.2021.609588 PMC7930621

[14] Lang M Moore NZ Rasmussen PA. Treatment outcomes of a randomized trial of unruptured brain arteriovenous malformation-eligible unruptured brain arteriovenous malformation patients. Neurosurgery 2018;83:548–555. 29040773 10.1093/neuros/nyx506

[15] Abecassis IJ Nerva JD Feroze A. Multimodality management of Spetzler-Martin grade 3 brain arteriovenous malformations with subgroup analysis. World Neurosurg 2017;102:263–274. 28323192 10.1016/j.wneu.2017.03.046

[16] Tong X Wu J Lin F. Brain arteriovenous malformations in elderly patients: clinical features and treatment outcome. Acta Neurochir (Wien) 2015;157:1645–1653. 26276468 10.1007/s00701-015-2521-6

[17] Nerva JD Mantovani A Barber J. Treatment outcomes of unruptured arteriovenous malformations with a subgroup analysis of ARUBA (a randomized trial of unruptured brain arteriovenous malformations)-eligible patients. Neurosurgery 2015;76:563–570. 25635891 10.1227/NEU.0000000000000663

[18] Chen Y Han H Ma L. Multimodality treatment for brain arteriovenous malformation in Mainland China: design, rationale, and baseline patient characteristics of a nationwide multicenter prospective registry. Chin Neurosurg J 2022;8:33. 36253875 10.1186/s41016-022-00296-y PMC9575306

[19] Chen Y Han H Jin H. Association of embolization with long-term outcomes in brain arteriovenous malformations: a propensity score-matched analysis using nationwide multicenter prospective registry data. Int J Surg 2023;109:1900–1909. 37226884 10.1097/JS9.0000000000000341 PMC10389468

[20] Chen Y Han H Meng X. Development and validation of a scoring system for hemorrhage risk in brain arteriovenous malformations. JAMA Netw Open 2023;6:e231070. 36857052 10.1001/jamanetworkopen.2023.1070 PMC9978947

[21] Austin PC Xin Yu AY Vyas MV. Applying propensity score methods in clinical research in neurology. Neurology 2021;97:856–863. 34504033 10.1212/WNL.0000000000012777 PMC8610625

[22] Mathew G Agha R Albrecht J. STROCSS 2021: strengthening the reporting of cohort, cross-sectional and case-control studies in surgery. Int J Surg 2021;96:106165. 34774726 10.1016/j.ijsu.2021.106165

[23] Atkinson RP Awad IA Batjer HH. Reporting terminology for brain arteriovenous malformation clinical and radiographic features for use in clinical trials. Stroke 2001;32:1430–1442. 11387510 10.1161/01.str.32.6.1430

[24] Psaty BM Siscovick DS. Minimizing bias due to confounding by indication in comparative effectiveness research: the importance of restriction. JAMA 2010;304:897–898. 20736474 10.1001/jama.2010.1205

[25] Kyriacou DN Lewis RJ. Confounding by indication in clinical research. JAMA 2016;316:1818–1819. 27802529 10.1001/jama.2016.16435

[26] VanderWeele TJ Ding P. Sensitivity analysis in observational research: introducing the E-Value. Ann Intern Med 2017;167:268. 28693043 10.7326/M16-2607

[27] Sattari SA Shahbandi A Yang W. Microsurgery versus microsurgery with preoperative embolization for brain arteriovenous malformation treatment: a systematic review and meta-analysis. Neurosurgery 2023;92:27–41. 36519858 10.1227/neu.0000000000002171

[28] Brosnan C Amoo M Javadpour M. Preoperative embolisation of brain arteriovenous malformations: a systematic review and meta-analysis. Neurosurg Rev 2022;45:2051–2063. 35260972 10.1007/s10143-022-01766-8 PMC9160113

[29] Letchuman V Mittal AM Gupta HR. The era of onyx embolization: a systematic and literature review of preoperative embolization before stereotactic radiosurgery for the management of cerebral arteriovenous malformations. World Neurosurg 2023;170:90–98. 36396047 10.1016/j.wneu.2022.11.058

[30] Hasegawa T Kato T Naito T. Effect of embolization before stereotactic radiosurgery for brain arteriovenous malformations: a case-control study with propensity score matching. *J Neurosurg*. Published online September 1, 2022:1-7. 10.3171/2022.7.JNS221343 36087321

[31] Hasegawa T Kato T Naito T. Long-term risks of hemorrhage and adverse radiation effects of stereotactic radiosurgery for brain arteriovenous malformations. Neurosurgery 2022;90:784–792. 35315812 10.1227/neu.0000000000001913

[32] Derdeyn CP Zipfel GJ Albuquerque FC. Management of brain arteriovenous malformations: a scientific statement for healthcare professionals from the American Heart Association/American Stroke Association. Stroke 2017;48:e200–e224. 28642352 10.1161/STR.0000000000000134

[33] Friedlander RM. Clinical practice. Arteriovenous malformations of the brain. N Engl J Med 2007;356:2704–2712. 17596605 10.1056/NEJMcp067192

[34] Pollock BE Flickinger JC Lunsford LD. Hemorrhage risk after stereotactic radiosurgery of cerebral arteriovenous malformations. Neurosurgery 1996;38:652–659. 8692381

[35] Nataf F Ghossoub M Schlienger M. Bleeding after radiosurgery for cerebral arteriovenous malformations. Neurosurgery 2004;55:298–305. 15271235 10.1227/01.neu.0000129473.52172.b5

[36] Al-Shahi Salman R White PM Counsell CE. Outcome after conservative management or intervention for unruptured brain arteriovenous malformations. JAMA 2014;311:1661–1669. 24756516 10.1001/jama.2014.3200

[37] Mohr JP Parides MK Stapf C. Medical management with or without interventional therapy for unruptured brain arteriovenous malformations (ARUBA): a multicentre, non-blinded, randomised trial. Lancet 2014;383:614–621. 24268105 10.1016/S0140-6736(13)62302-8 PMC4119885

[38] Ding D Starke RM Kano H. Stereotactic radiosurgery for Spetzler-Martin Grade III arteriovenous malformations: an international multicenter study. J Neurosurg 2017;126:859–871. 27081906 10.3171/2016.1.JNS152564

[39] Starke RM Kano H Ding D. Stereotactic radiosurgery for cerebral arteriovenous malformations: evaluation of long-term outcomes in a multicenter cohort. J Neurosurg 2017;126:36–44. 26943847 10.3171/2015.9.JNS151311

[40] Kim H Abla AA Nelson J. Validation of the supplemented Spetzler-Martin grading system for brain arteriovenous malformations in a multicenter cohort of 1009 surgical patients. Neurosurgery 2015;76:25–31. 25251197 10.1227/NEU.0000000000000556 PMC4270816

[41] OʼConnor TE Friedman WA. Magnetic resonance imaging assessment of cerebral arteriovenous malformation obliteration after stereotactic radiosurgery. Neurosurgery 2013;73:761–766. 23863762 10.1227/NEU.0000000000000086

[42] Lee CC Reardon MA Ball BZ. The predictive value of magnetic resonance imaging in evaluating intracranial arteriovenous malformation obliteration after stereotactic radiosurgery. J Neurosurg 2015;123:136–144. 25839923 10.3171/2014.10.JNS141565

