# Supplementary material for: Long-term outcomes of microsurgery and stereotactic radiosurgery as the first-line treatment for arteriovenous malformations: a propensity score-matched analysis using nationwide multicenter prospective registry data
Source: Int J Surg. 2023 Sep 14;109(12):3983–92. doi: 10.1097/JS9.0000000000000751 (PMC10720861; doi:10.1097/JS9.0000000000000751)
Supplement: SUPPLEMENTARY MATERIAL [file js9-109-3983-s001.docx]

Content

[**Supplementary Method 1. Protocol for data quality management.** 2](#_Toc141646652)

[**Supplementary Method 2. Measures for reducing follow-up bias.** 3](#_Toc141646653)

[**Supplementary Table 1. Breakdown of missing data before propensity score matching.** 4](#_Toc141646654)

[**Supplementary Table 2. Comparisons of characteristics in patients included and lost to follow-up.** 5](#_Toc141646655)

[**Supplementary Figure 1. Balance assessment of the propensity score matching in unruptured, ruptured and overall AVMs.** 9](#_Toc141646656)

[**Supplementary Table 4. Baseline characteristics before and after propensity score matching.** 11](#_Toc141646657)

[**Supplementary Table 5. Follow-up and censoring reasons after propensity score matching (primary outcomes).** 17](#_Toc141646658)

[**Supplementary Figure 2. Subgroup analyses for neurofunctional outcomes.** 18](#_Toc141646659)

[**Supplementary Table 6. Subgroup analyses for hemorrhagic stroke or death categorized by S-M grade.** 19](#_Toc141646660)

[**Supplementary Table 7. Subgroup analyses for disabling functional outcomes categorized by S-M grade.** 20](#_Toc141646661)

[**Supplementary Table 8. Subgroup analyses for neurofunctional decline categorized by S-M grade and Supp S-M grade.** 21](#_Toc141646662)

[**Supplementary Table 9. Sensitivity analyses with different study designs.** 22](#_Toc141646663)

**Supplementary Method 1. Protocol for data quality management.**

1. Definition of variables were discussed and unified according to the terminology reporting standards or published paper before the initiation of data collection. Clinical research coordinators (CRCs) and neurosurgery residents were then trained by cerebrovascular neurosurgeons with more than 15 years’ working experience. CRCs were responsible for demographic information and follow-up data, and neurosurgery residents for angiographic features. The two parts were blinded to each other to ensure the data collected were not biased by imaging characteristics or clinical outcomes.
2. A standard training dataset with 50 cases were used to check the consistency of data collectors. For those variables or cases with significant interobserver variation, the consensus was reached by either modifying the confusing definitions or retraining the data collectors. Only when the consistency reached 90% can the CRC or the resident allowed to extracting information independently.
3. While recording data, one could ask for help about unsure cases in a discussion group with cerebrovascular neurosurgeons in it, or mark these cases and discuss in weekly meetings.
4. The group leader with more than five years’ working experience randomly spot checks these data biweekly. Investigators would receive training again if their data were of low quality, and these data would be recollected by other investigators.

**Supplementary Method 2. Measures for reducing follow-up bias.**

1. A follow-up schedule is established at the beginning of the study. Patients are informed of regular follow-up visits at 3 months, annually (1, 2, and 3 years), and every 5 years after the treatment.
2. To increase participant retention, we aim to build a rapport with participants from the outset. We build online chatgroups for the convenience of doctor-patient and patient-patient communication. In this online community, we explain the purpose and importance of the study, answer their questions, and provide regular updates about the study's progress. We also provide feedback on their contribution to the study and share relevant results (where appropriate). A telemedicine applet (*BLINDED*) is also used for online consultation and follow-up.
3. In cases where participants do not respond to follow-up attempts, we implement a standardized protocol for additional contact attempts. This involves using alternate contact methods or reaching out to outpatient medical records. If these attempts turned out to be unsuccessful, the participant is classified as lost to follow-up.
4. Despite our best efforts, some participants may be lost to follow-up. We regularly compare characteristics in patients included and lost to follow-up according to different study designs. Similar characteristics would imply the rationale for analyzing the included data with relatively low follow-up bias.
5. Similar to data collection, the follow-up process is reviewed periodically by the group leader. Any issues or areas for improvement are identified and addressed. Refresher training sessions are conducted as needed to ensure that clinical research coordinator members are up-to-date on the follow-up protocol and best practices.

**Supplementary Table 1. Breakdown of missing data before propensity score matching.**

|  | Unruptured AVMs | | Ruptured AVMs | |
| --- | --- | --- | --- | --- |
| Characteristics | MS | SRS | MS | SRS |
| No. of patients | 389 | 234 | 576 | 405 |
| Sex | - | - | - | - |
| Age at diagnosis | - | - | - | - |
| Admission mRS | - | - | - | - |
| Seizure | - | - | - | - |
| Neurological deficiency | - | - | - | - |
| Spetzler-Martin grade | 9 (2.3) | 2 (0.9) | 21 (3.6) | 3 (0.7) |
| Size | - | - | - | - |
| Location | - | - | - | - |
| Frontal lobe | - | - | - | - |
| Temporal lobe | - | - | - | - |
| Parietal lobe | - | - | - | - |
| Occipital lobe | - | - | - | - |
| Basal ganglia | - | - | - | - |
| Cerebellum | - | - | - | - |
| Brain stem | - | - | - | - |
| SRS margin dose, Gy (SD) | - | - | - | - |
| SRS central dose, Gy (SD) | - | - | - | - |
| Ventricular system involvement | - | - | - | - |
| Eloquent region | - | - | - | - |
| Feeding artery |  |  |  |  |
| Single feeder | 14 (3.6) | 2 (0.9) | 32 (5.6) | 2 (0.5) |
| Multiple sources | 11 (2.8) | 2 (0.9) | 20 (3.5) | 2 (0.5) |
| Perforating artery | 14 (3.6) | 1 (0.4) | 28 (4.9) | 3 (0.7) |
| Flow-related aneurysm | 10 (2.6) | 4 (1.7) | 24 (4.2) | 3 (0.7) |
| Diffuse nidus | 13 (3.3) | - | 38 (6.6) | 1 (0.2) |
| Venous draining |  |  |  |  |
| Stenosis | 13 (3.3) | 4 (1.7) | 21 (3.6) | 3 (0.7) |
| Any deep drainage | 9 (2.3) | 2 (0.9) | 21 (3.6) | 3 (0.7) |
| Exclusively deep drainage | 12 (3.1) | - | 20 (3.5) | 1 (0.2) |
| Venous aneurysm | 11 (2.8) | 1 (0.4) | 26 (4.5) | 2 (0.5) |

Abbreviation: AVM, arteriovenous malformation; mRS, modified Rankin Scale; MS, microsurgery; SD, standard deviation; SRS, stereotactic radiosurgery.

**Supplementary Table 2. Comparisons of characteristics in patients included and lost to follow-up.**

|  | Unruptured AVMs | |  | Ruptured AVMs | |  |
| --- | --- | --- | --- | --- | --- | --- |
| Characteristics | Included | Lost to follow-up | P | Included | Lost to follow-up | P |
| No. of patients | 519 | 104 |  | 829 | 152 |  |
| Sex (female) | 197 (38.0) | 32 (30.8) | 0.202 | 353 (42.6) | 62 (40.8) | 0.748 |
| Age at diagnosis, mean (SD) | 27.67 (14.23) | 27.61 (13.77) | 0.965 | 23.72 (14.34) | 23.36 (14.57) | 0.778 |
| Admission mRS, median (IQR) | 1.00 [1.00, 1.00] | 1.00 [1.00, 1.00] | 0.643 | 1.00 [1.00, 2.00] | 1.00 [1.00, 2.00] | 0.606 |
| Seizure | 231 (44.5) | 46 (44.2) | >0.999 | 111 (13.4) | 26 (17.1) | 0.277 |
| Neurological deficiency | 74 (14.3) | 17 (16.3) | 0.690 | 210 (25.3) | 29 (19.1) | 0.122 |
| S-M grade |  |  | 0.569 |  |  | 0.210 |
| I-II | 282 (54.3) | 55 (52.9) |  | 488 (58.9) | 78 (51.3) |  |
| III | 172 (33.1) | 39 (37.5) |  | 247 (29.8) | 55 (36.2) |  |
| IV-V | 65 (12.5) | 10 (9.6) |  | 94 (11.3) | 19 (12.5) |  |
| Size, cm |  |  | 0.688 |  |  | 0.562 |
| <3 | 216 (41.6) | 48 (46.2) |  | 560 (67.6) | 96 (63.2) |  |
| 3-6 | 263 (50.7) | 49 (47.1) |  | 243 (29.3) | 51 (33.6) |  |
| >6 | 40 (7.7) | 7 (6.7) |  | 26 (3.1) | 5 (3.3) |  |
| Location |  |  |  |  |  |  |
| Frontal lobe | 181 (34.9) | 31 (29.8) | 0.378 | 167 (20.1) | 36 (23.7) | 0.378 |
| Temporal lobe | 158 (30.4) | 33 (31.7) | 0.886 | 239 (28.8) | 36 (23.7) | 0.230 |
| Parietal lobe | 116 (22.4) | 25 (24.0) | 0.805 | 190 (22.9) | 44 (28.9) | 0.134 |
| Occipital lobe | 90 (17.3) | 25 (24.0) | 0.142 | 148 (17.9) | 24 (15.8) | 0.618 |
| Basal ganglia | 36 (6.9) | 7 (6.7) | >0.999 | 112 (13.5) | 22 (14.5) | 0.850 |
| Cerebellum | 22 (4.2) | 2 (1.9) | 0.400 | 78 (9.4) | 10 (6.6) | 0.333 |
| Brain stem | 15 (2.9) | 3 (2.9) | >0.999 | 31 (3.7) | 6 (3.9) | >0.999 |
| Ventricular system involvement | 175 (33.7) | 35 (33.7) | >0.999 | 545 (65.7) | 103 (67.8) | 0.696 |
| Eloquent region | 250 (48.2) | 52 (50.0) | 0.815 | 470 (56.7) | 95 (62.5) | 0.214 |
| Feeding artery |  |  |  |  |  |  |
| Single feeder | 110 (21.2) | 23 (22.1) | 0.938 | 401 (48.4) | 63 (41.4) | 0.138 |
| Multiple sources | 156 (30.1) | 33 (31.7) | 0.824 | 150 (18.1) | 31 (20.4) | 0.577 |
| Perforating artery | 136 (26.2) | 21 (20.2) | 0.244 | 329 (39.7) | 48 (31.6) | 0.072 |
| Flow-related aneurysm | 70 (13.5) | 9 (8.7) | 0.234 | 131 (15.8) | 25 (16.4) | 0.937 |
| Diffuse nidus | 108 (20.8) | 16 (15.4) | 0.258 | 372 (44.9) | 67 (44.1) | 0.926 |
| Venous draining |  |  |  |  |  |  |
| Stenosis | 65 (12.5) | 14 (13.5) | 0.920 | 136 (16.4) | 25 (16.4) | >0.999 |
| Any deep drainage | 139 (26.8) | 26 (25.0) | 0.799 | 363 (43.8) | 63 (41.4) | 0.656 |
| Exclusively deep drainage | 70 (13.5) | 11 (10.6) | 0.518 | 283 (34.1) | 50 (32.9) | 0.838 |
| Venous aneurysm | 141 (27.2) | 32 (30.8) | 0.530 | 41 (4.9) | 10 (6.6) | 0.525 |

Abbreviation: AVM, arteriovenous malformation; IQR, interquartile range; mRS, modified Rankin Scale; MS, microsurgery; SD, standard deviation; S-M grade, Spetzler-Martin grade; SRS, stereotactic radiosurgery.

**Supplementary Table 3. Number and proportional (%) breakdown of characteristics in patients lost to follow-up.**

|  | Unruptured AVMs | |  | Ruptured AVMs | |  |
| --- | --- | --- | --- | --- | --- | --- |
| Characteristics | MS | SRS | P | MS | SRS | P |
| No. of patients | 60 | 44 |  | 86 | 66 |  |
| Sex (female) | 18 (30.0) | 14 (31.8) | >0.999 | 31 (36.0) | 31 (47.0) | 0.233 |
| Age at diagnosis, mean (SD) | 25.9 (12.9) | 30.0 (14.7) | 0.129 | 24.2 (16.1) | 22.3 (12.4) | 0.438 |
| Admission mRS, median (IQR) | 1.00 [0.75, 1.00] | 1.00 [1.00, 1.00] | 0.396 | 1.00 [1.00, 2.00] | 1.00 [0.00, 2.00] | 0.035 |
| Seizure | 31 (51.7) | 15 (34.1) | 0.113 | 14 (16.3) | 12 (18.2) | 0.927 |
| Neurological deficiency | 8 (13.3) | 9 (20.5) | 0.483 | 19 (22.1) | 10 (15.2) | 0.384 |
| S-M grade |  |  | 0.571 |  |  | 0.445 |
| I-II | 30 (50.0) | 25 (56.8) |  | 48 (55.8) | 30 (45.5) |  |
| III | 25 (41.7) | 14 (31.8) |  | 28 (32.6) | 27 (40.9) |  |
| IV-V | 5 (8.3) | 5 (11.4) |  | 10 (11.6) | 9 (13.6) |  |
| Size, cm |  |  | <0.001 |  |  | 0.158 |
| <3 | 17 (28.3) | 31 (70.5) |  | 49 (57.0) | 47 (71.2) |  |
| 3-6 | 38 (63.3) | 11 (25.0) |  | 33 (38.4) | 18 (27.3) |  |
| >6 | 5 (8.3) | 2 (4.5) |  | 4 (4.7) | 1 (1.5) |  |
| Location |  |  |  |  |  |  |
| Frontal lobe | 26 (43.3) | 5 (11.4) | 0.001 | 27 (31.4) | 9 (13.6) | 0.018 |
| Temporal lobe | 25 (41.7) | 8 (18.2) | 0.020 | 24 (27.9) | 12 (18.2) | 0.228 |
| Parietal lobe | 11 (18.3) | 14 (31.8) | 0.175 | 27 (31.4) | 17 (25.8) | 0.562 |
| Occipital lobe | 14 (23.3) | 11 (25.0) | >0.999 | 21 (24.4) | 3 (4.5) | 0.002 |
| Basal ganglia | 0 (0.0) | 2 (4.5) | 0.345 | 7 (8.1) | 3 (4.5) | 0.578 |
| Cerebellum | 0 (0.0) | 3 (6.8) | 0.144 | 0 (0.0) | 6 (9.1) | 0.015 |
| Brain stem | 0 (0.0) | 7 (15.9) | 0.005 | 5 (5.8) | 17 (25.8) | 0.001 |
| Ventricular system involvement | 13 (21.7) | 22 (50.0) | 0.005 | 50 (58.1) | 53 (80.3) | 0.006 |
| Eloquent region | 26 (43.3) | 26 (59.1) | 0.165 | 50 (58.1) | 45 (68.2) | 0.272 |
| Feeding artery |  |  |  |  |  |  |
| Single feeder | 13 (21.7) | 10 (22.7) | >0.999 | 31 (36.0) | 32 (48.5) | 0.169 |
| Multiple sources | 24 (40.0) | 9 (20.5) | 0.057 | 21 (24.4) | 10 (15.2) | 0.229 |
| Perforating artery | 7 (11.7) | 14 (31.8) | 0.022 | 14 (16.3) | 34 (51.5) | <0.001 |
| Flow-related aneurysm | 5 (8.3) | 4 (9.1) | >0.999 | 21 (24.4) | 4 (6.1) | 0.005 |
| Diffuse nidus | 10 (16.7) | 6 (13.6) | 0.882 | 32 (37.2) | 35 (53.0) | 0.075 |
| Venous draining |  |  |  |  |  |  |
| Stenosis | 11 (18.3) | 3 (6.8) | 0.159 | 16 (18.6) | 9 (13.6) | 0.550 |
| Any deep drainage | 11 (18.3) | 15 (34.1) | 0.109 | 23 (26.7) | 40 (60.6) | <0.001 |
| Exclusively deep drainage | 3 (5.0) | 8 (18.2) | 0.066 | 14 (16.3) | 36 (54.5) | <0.001 |
| Venous aneurysm | 24 (40.0) | 8 (18.2) | 0.030 | 7 (8.1) | 3 (4.5) | 0.578 |

Abbreviation: AVM, arteriovenous malformation; IQR, interquartile range; mRS, modified Rankin Scale; MS, microsurgery; SD, standard deviation; S-M grade, Spetzler-Martin grade; SRS, stereotactic radiosurgery.

**Supplementary Figure 1. Balance assessment of the propensity score matching in unruptured, ruptured and overall AVMs.**

1. Unruptured AVMs


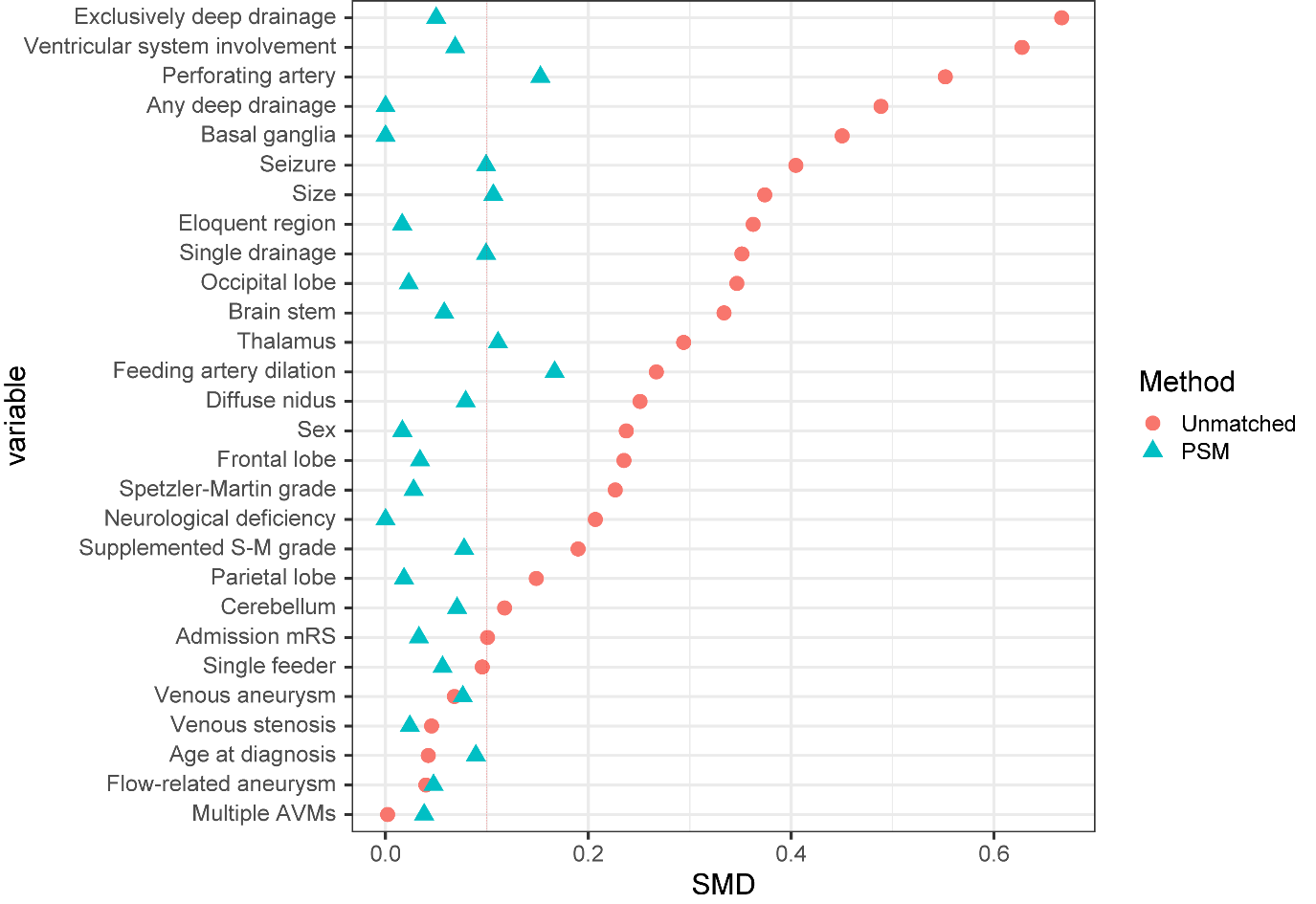


1. Ruptured AVMs


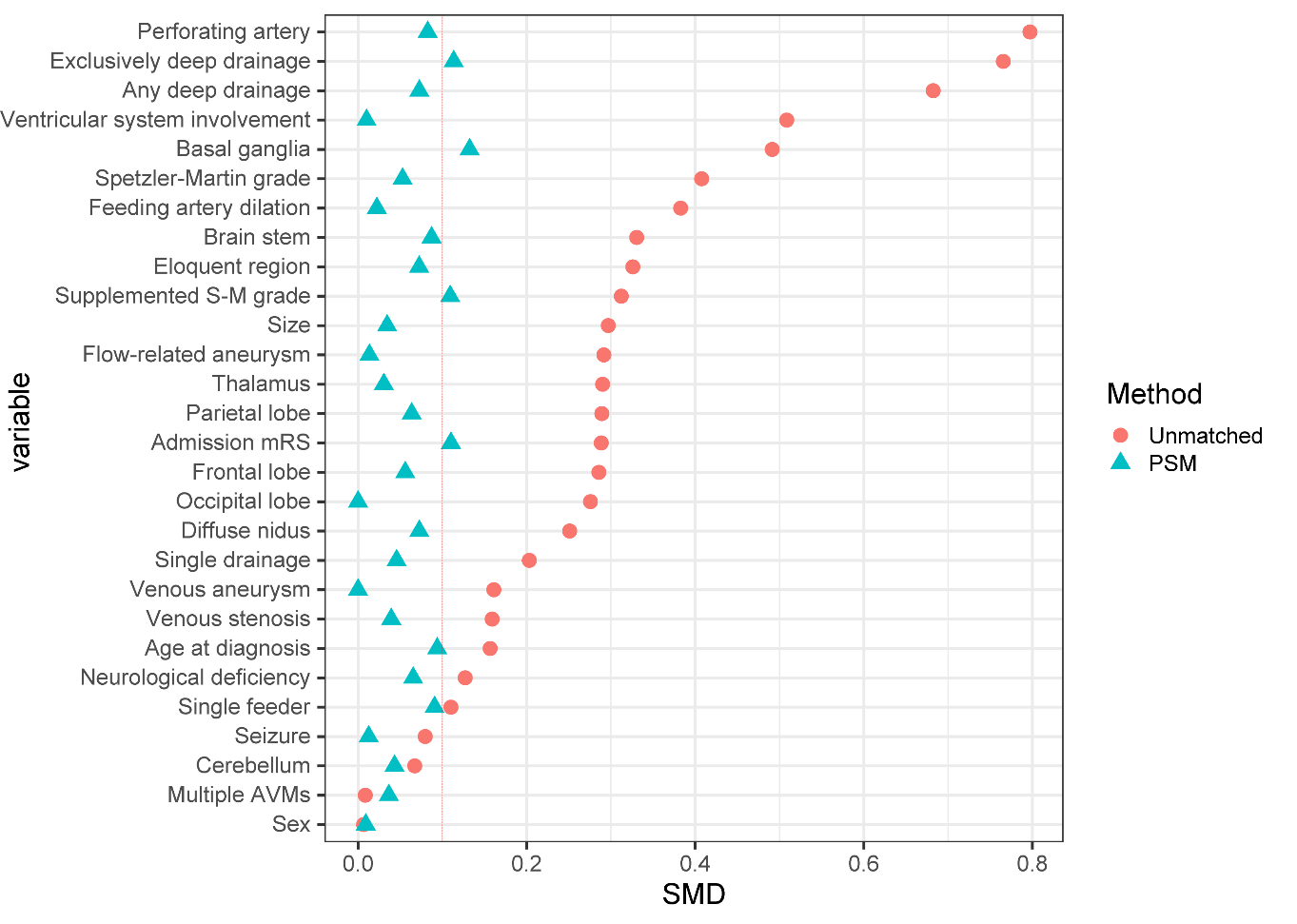


1. Overall population


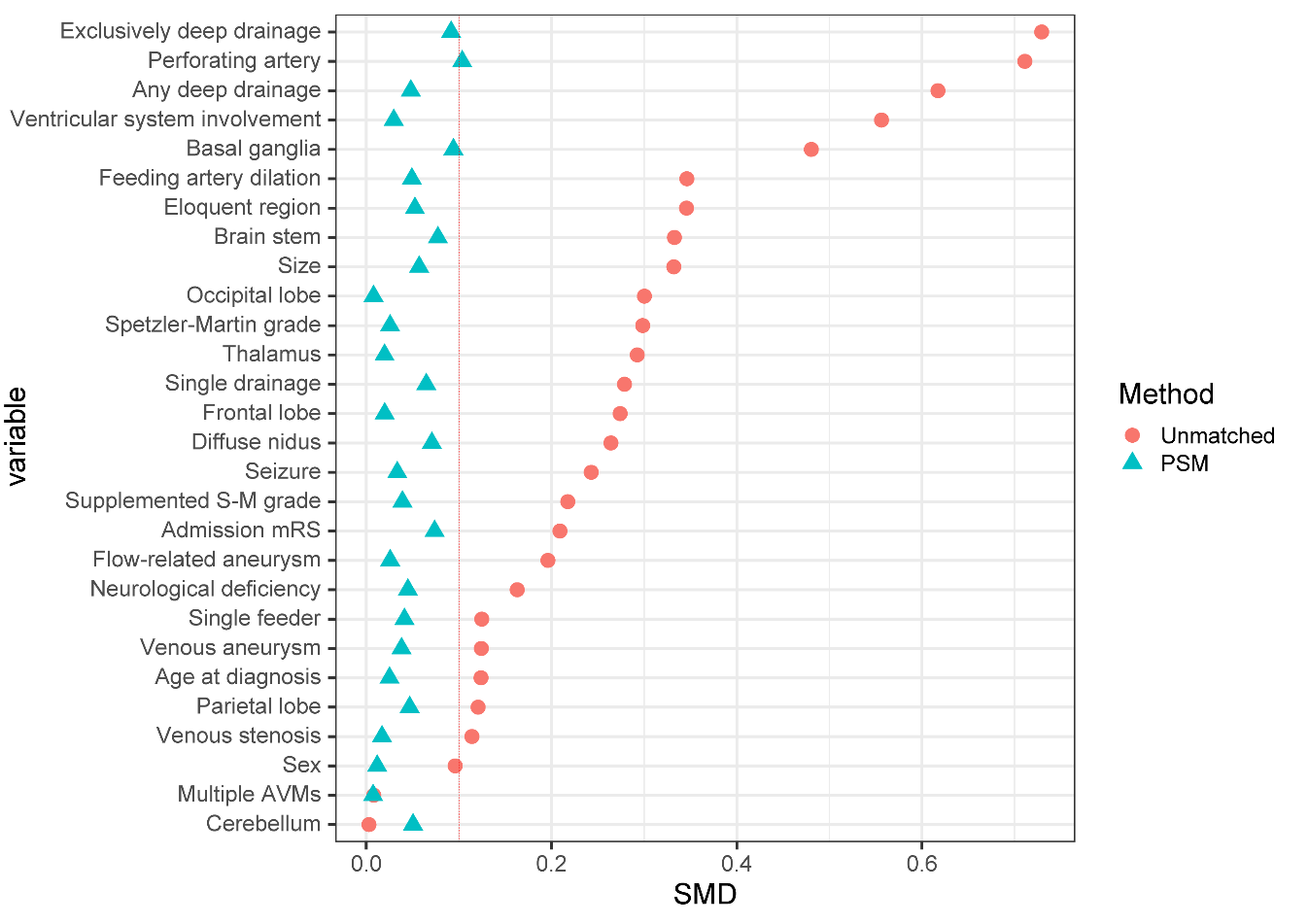


**Supplementary Table 4. Baseline characteristics before and after propensity score matching.**

| Characteristics before PSM | Unruptured | | | Ruptured | | | Overall | | |
| --- | --- | --- | --- | --- | --- | --- | --- | --- | --- |
|  | MS | SRS | *P* | MS | SRS | *P* | MS | SRS | *P* |
| No. of patients | 329 | 190 |  | 490 | 339 |  | 819 | 529 |  |
| Sex (female) | 111 (33.7) | 86 (45.3) | 0.012 | 208 (42.4) | 145 (42.8) | 0.983 | 319 (38.9) | 231 (43.7) | 0.096 |
| Age at diagnosis, mean (SD) | 27.9 (13.7) | 27.3 (15.2) | 0.639 | 24.6 (15.1) | 22.4 (13.1) | 0.029 | 25.9 (14.6) | 24.2 (14.0) | 0.027 |
| Admission mRS, median (IQR) | 1 [1, 1] | 1 [1, 1] | 0.128 | 1 [1, 2] | 1 [0, 2] | <0.001 | 1 [1, 2] | 1 [0, 1] | <0.001 |
| Seizure | 170 (51.7) | 61 (32.1) | <0.001 | 71 (14.5) | 40 (11.8) | 0.310 | 241 (29.4) | 101 (19.1) | <0.001 |
| Neurological deficiency | 38 (11.6) | 36 (18.9) | 0.028 | 113 (23.1) | 97 (28.6) | 0.084 | 151 (18.4) | 133 (25.1) | 0.004 |
| Spetzler-Martin grade |  |  | 0.039 |  |  | <0.001 |  |  | <0.001 |
| I-II | 186 (56.5) | 96 (50.5) |  | 328 (66.9) | 160 (47.2) |  | 514 (62.8) | 256 (48.4) |  |
| III | 111 (33.7) | 61 (32.1) |  | 116 (23.7) | 131 (38.6) |  | 227 (27.7) | 192 (36.3) |  |
| IV-V | 32 (9.7) | 33 (17.4) |  | 46 (9.4) | 48 (14.2) |  | 78 (9.5) | 81 (15.3) |  |
| Size, cm |  |  | <0.001 |  |  | <0.001 |  |  | <0.001 |
| <3 | 115 (35.0) | 101 (53.2) |  | 305 (62.2) | 255 (75.2) |  | 420 (51.3) | 356 (67.3) |  |
| 3-6 | 185 (56.2) | 78 (41.1) |  | 170 (34.7) | 73 (21.5) |  | 355 (43.3) | 151 (28.5) |  |
| >6 | 29 (8.8) | 11 (5.8) |  | 15 (3.1) | 11 (3.2) |  | 44 (5.4) | 22 (4.2) |  |
| Location |  |  |  |  |  |  |  |  |  |
| Frontal lobe | 128 (38.9) | 53 (27.9) | 0.015 | 121 (24.7) | 46 (13.6) | <0.001 | 249 (30.4) | 99 (18.7) | <0.001 |
| Temporal lobe | 116 (35.3) | 42 (22.1) | 0.002 | 167 (34.1) | 72 (21.2) | <0.001 | 283 (34.6) | 114 (21.6) | <0.001 |
| Parietal lobe | 66 (20.1) | 50 (26.3) | 0.124 | 136 (27.8) | 54 (15.9) | <0.001 | 202 (24.7) | 104 (19.7) | 0.038 |
| Occipital lobe | 72 (21.9) | 18 (9.5) | 0.001 | 108 (22.0) | 40 (11.8) | <0.001 | 180 (22.0) | 58 (11.0) | <0.001 |
| Basal ganglia | 8 (2.4) | 28 (14.7) | <0.001 | 32 (6.5) | 80 (23.6) | <0.001 | 61 (7.4) | 39 (7.4) | >0.999 |
| Cerebellum | 11 (3.3) | 11 (5.8) | 0.269 | 50 (10.2) | 28 (8.3) | 0.411 | 7 (0.9) | 39 (7.4) | <0.001 |
| Brain stem | 2 (0.6) | 13 (6.8) | <0.001 | 5 (1.0) | 26 (7.7) | <0.001 | 40 (4.9) | 108 (20.4) | <0.001 |
| SRS margin dose, Gy (SD) | - | 16.0 [15.0, 17.0] | - | - | 17.0 [16.0, 18.0] | - | - | 17.0 [16.0, 18.0] | - |
| SRS central dose, Gy (SD) | - | 32.0 [30.0, 34.0] | - | - | 34.0 [32.0, 36.0] | - | - | 32.7 [30.0, 36.0] | - |
| Ventricular system involvement | 76 (23.1) | 99 (52.1) | <0.001 | 276 (56.3) | 269 (79.4) | <0.001 | 352 (43.0) | 368 (69.6) | <0.001 |
| Eloquent region | 137 (41.6) | 113 (59.5) | <0.001 | 246 (50.2) | 224 (66.1) | <0.001 | 383 (46.8) | 337 (63.7) | <0.001 |
| Feeding artery |  |  |  |  |  |  |  |  |  |
| Single feeder | 65 (19.8) | 45 (23.7) | 0.346 | 226 (46.1) | 175 (51.6) | 0.137 | 291 (35.5) | 220 (41.6) | 0.029 |
| Multiple sources | 99 (30.1) | 57 (30.0) | >0.999 | 88 (18.0) | 62 (18.3) | 0.976 | 187 (22.8) | 119 (22.5) | 0.938 |
| Perforating artery | 57 (17.3) | 79 (41.6) | <0.001 | 121 (24.7) | 208 (61.4) | <0.001 | 178 (21.7) | 287 (54.3) | <0.001 |
| Flow-related aneurysm | 46 (14.0) | 24 (12.6) | 0.764 | 98 (20.0) | 33 (9.7) | <0.001 | 144 (17.6) | 57 (10.8) | 0.001 |
| Diffuse nidus | 56 (17.0) | 52 (27.4) | 0.007 | 195 (39.8) | 177 (52.2) | 0.001 | 251 (30.6) | 229 (43.3) | <0.001 |
| Venous draining |  |  |  |  |  |  |  |  |  |
| Stenosis | 43 (13.1) | 22 (11.6) | 0.721 | 92 (18.8) | 44 (13.0) | 0.034 | 135 (16.5) | 66 (12.5) | 0.053 |
| Any deep drainage | 62 (18.8) | 77 (40.5) | <0.001 | 150 (30.6) | 213 (62.8) | <0.001 | 212 (25.9) | 290 (54.8) | <0.001 |
| Exclusively deep drainage | 16 (4.9) | 54 (28.4) | <0.001 | 98 (20.0) | 185 (54.6) | <0.001 | 114 (13.9) | 239 (45.2) | <0.001 |
| Venous aneurysm | 93 (28.3) | 48 (25.3) | 0.523 | 31 (6.3) | 10 (2.9) | 0.041 | 124 (15.1) | 58 (11.0) | 0.035 |
| Characteristics after PSM | Unruptured | | | Ruptured | | | Overall | | |
|  | MS | SRS | *P* | MS | SRS | *P* | MS | SRS | *P* |
| No. of patients | 122 | 122 |  | 221 | 221 |  | 343 | 343 |  |
| Sex (male) | 51 (41.8) | 52 (42.6) | >0.999 | 96 (43.4) | 97 (43.9) | >0.999 | 147 (42.9) | 149 (43.4) | 0.939 |
| Age at diagnosis, mean (SD) | 28.9 (13.3) | 27.7 (15.0) | 0.487 | 20.8 (13.4) | 22.1 (13.1) | 0.324 | 23.7 (13.9) | 24.1 (14.0) | 0.742 |
| Admission mRS, median (IQR) | 1 [1, 1] | 1 [1, 1] | 0.788 | 1 [1, 2] | 1 [1, 2] | 0.363 | 1 [1, 1] | 1 [1, 1] | 0.536 |
| Seizure | 57 (46.7) | 51 (41.8) | 0.519 | 33 (14.9) |  | >0.999 | 90 (26.2) | 85 (24.8) | 0.726 |
| Neurological deficiency | 15 (12.3) | 15 (12.3) | >0.999 | 46 (20.8) | 52 (23.5) | 0.567 | 61 (17.8) | 67 (19.5) | 0.624 |
| Spetzler-Martin grade |  |  | 0.977 |  |  | 0.857 |  |  | 0.945 |
| I-II | 73 (59.8) | 74 (60.7) |  | 144 (65.2) | 139 (62.9) |  | 217 (63.3) | 213 (62.1) |  |
| III | 36 (29.5) | 36 (29.5) |  | 54 (24.4) | 56 (25.3) |  | 90 (26.2) | 92 (26.8) |  |
| IV-V | 13 (10.7) | 12 (9.8) |  | 23 (10.4) | 26 (11.8) |  | 36 (10.5) | 38 (11.1) |  |
| Size, cm |  |  | 0.709 |  |  | 0.936 |  |  | 0.756 |
| <3 | 63 (51.6) | 63 (51.6) |  | 157 (71.0) | 160 (72.4) |  | 220 (64.1) | 223 (65.0) |  |
| 3-6 | 50 (41.0) | 53 (43.4) |  | 56 (25.3) | 54 (24.4) |  | 106 (30.9) | 107 (31.2) |  |
| >6 | 9 (7.4) | 6 (4.9) |  | 8 (3.6) | 7 (3.2) |  | 17 (5.0) | 13 (3.8) |  |
| Location |  |  |  |  |  |  |  |  |  |
| Frontal lobe | 44 (36.1) | 46 (37.7) | 0.894 | 48 (21.7) | 43 (19.5) | 0.638 | 92 (26.8) | 89 (25.9) | 0.862 |
| Temporal lobe | 36 (29.5) | 30 (24.6) | 0.471 | 58 (26.2) | 61 (27.6) | 0.830 | 94 (27.4) | 91 (26.5) | 0.863 |
| Parietal lobe | 35 (28.7) | 34 (27.9) | >0.999 | 56 (25.3) | 50 (22.6) | 0.578 | 91 (26.5) | 84 (24.5) | 0.599 |
| Occipital lobe | 19 (15.6) | 18 (14.8) | >0.999 | 38 (17.2) | 38 (17.2) | >0.999 | 57 (16.6) | 56 (16.3) | >0.999 |
| Basal ganglia | 7 (5.7) | 7 (5.7) | >0.999 | 25 (11.3) | 35 (15.8) | 0.211 | 29 (8.5) | 34 (9.9) | 0.597 |
| Cerebellum | 6 (4.9) | 8 (6.6) | 0.783 | 23 (10.4) | 26 (11.8) | 0.762 | 6 (1.7) | 10 (2.9) | 0.448 |
| Brain stem | 2 (1.6) | 3 (2.5) | >0.999 | 4 (1.8) | 7 (3.2) | 0.541 | 32 (9.3) | 42 (12.2) | 0.268 |
| SRS margin dose, Gy (SD) | - | 16.0 [15.0, 17.0] | - | - | 17.0 [16.0, 18.0] | - | - | 17.0 [16.0, 18.0] | - |
| SRS central dose, Gy (SD) | - | 32.0 [30.0, 34.0] | - | - | 34.0 [32.0, 36.0] | - | - | 34.0 [31.8, 36.0] | - |
| Ventricular system involvement | 41 (33.6) | 45 (36.9) | 0.688 | 159 (71.9) | 160 (72.4) | >0.999 | 200 (58.3) | 205 (59.8) | 0.756 |
| Eloquent region | 60 (49.2) | 61 (50.0) | >0.999 | 111 (50.2) | 119 (53.8) | 0.505 | 171 (49.9) | 180 (52.5) | 0.541 |
| Feeding artery |  |  |  |  |  |  |  |  |  |
| Single feeder | 33 (27.0) | 30 (24.6) | 0.770 | 112 (50.7) | 122 (55.2) | 0.391 | 145 (42.3) | 152 (44.3) | 0.644 |
| Multiple sources | 29 (23.8) | 31 (25.4) | 0.882 | 38 (17.2) | 35 (15.8) | 0.798 | 67 (19.5) | 66 (19.2) | >0.999 |
| Perforating artery | 26 (21.3) | 34 (27.9) | 0.298 | 88 (39.8) | 97 (43.9) | 0.441 | 114 (33.2) | 131 (38.2) | 0.202 |
| Flow-related aneurysm | 18 (14.8) | 16 (13.1) | 0.853 | 29 (13.1) | 28 (12.7) | >0.999 | 47 (13.7) | 44 (12.8) | 0.822 |
| Diffuse nidus | 25 (20.5) | 29 (23.8) | 0.644 | 115 (52.0) | 123 (55.7) | 0.504 | 140 (40.8) | 152 (44.3) | 0.396 |
| Venous draining |  |  |  |  |  |  |  |  |  |
| Stenosis | 17 (13.9) | 16 (13.1) | >0.999 | 29 (13.1) | 32 (14.5) | 0.783 | 46 (13.4) | 48 (14.0) | 0.912 |
| Any deep drainage | 29 (23.8) | 29 (23.8) | >0.999 | 97 (43.9) | 105 (47.5) | 0.504 | 126 (36.7) | 134 (39.1) | 0.582 |
| Exclusively deep drainage | 14 (11.5) | 16 (13.1) | 0.845 | 73 (33.0) | 85 (38.5) | 0.275 | 87 (25.4) | 101 (29.4) | 0.266 |
| Venous aneurysm | 32 (26.2) | 28 (23.0) | 0.656 | 6 (2.7) | 6 (2.7) | >0.999 | 38 (11.1) | 34 (9.9) | 0.709 |

Abbreviation: AVM, arteriovenous malformation; IQR, interquartile range; mRS, modified Rankin Scale; MS, microsurgery; PSM, propensity score matching; SD, standard deviation; SRS, stereotactic radiosurgery.

**Supplementary Table 5. Follow-up and censoring reasons after propensity score matching (primary outcomes).**

|  | Unruptured AVMs | | | Ruptured AVMs | | | Overall population | | |
| --- | --- | --- | --- | --- | --- | --- | --- | --- | --- |
|  | Total | MS | SRS | Total | MS | SRS | Total | MS | SRS |
| No. of patients | 244 | 122 | 122 | 442 | 221 | 221 | 686 | 343 | 343 |
| Follow-up, median (IQR), y | 6.8 (3.9, 8.8) | 6.8 (4.4, 8.6) | 6.8 (3.3, 9.1) | 6.2 (3.0, 8.5) | 6.7 (3.4, 8.9) | 5.9 (2.9, 8.1) | 6.5 (3.3, 8.6) | 6.8 (3.9, 8.8) | 6.0 (3.1, 8.3) |
| Follow-up, mean (SD), y | 7.0 (4.4) | 6.9 (4.5) | 7.0 (4.3) | 6.1 (3.7) | 6.2 (3.5) | 6.0 (3.9) | 6.4 (4.0) | 6.4 (3.9) | 6.4 (4.0) |
| Censoring reasons (%) |  |  |  |  |  |  |  |  |  |
| Outcome occurrence | 16 (6.6) | 3 (2.5) | 13 (10.7) | 28 (6.3) | 5 (2.3) | 23 (10.4) | 44 (6.4) | 8 (2.3) | 36 (10.5) |
| Treatment switching | 23 (9.4) | 2 (1.6) | 21 (17.2) | 33 (7.5) | 18 (8.1) | 15 (6.8) | 56 (8.2) | 20 (5.8) | 36 (10.5) |
| Last follow-up | 205 (84.0) | 117 (95.9) | 88 (72.1) | 381 (86.2) | 198 (89.6) | 183 (82.8) | 586 (85.4) | 315 (91.8) | 271 (79.0) |

Abbreviations: AVM, arteriovenous malformation; IQR, interquartile range; MS, microsurgery; SD, standard deviation; SRS, stereotactic radiosurgery.

**Supplementary Figure 2. Subgroup analyses for neurofunctional outcomes.**


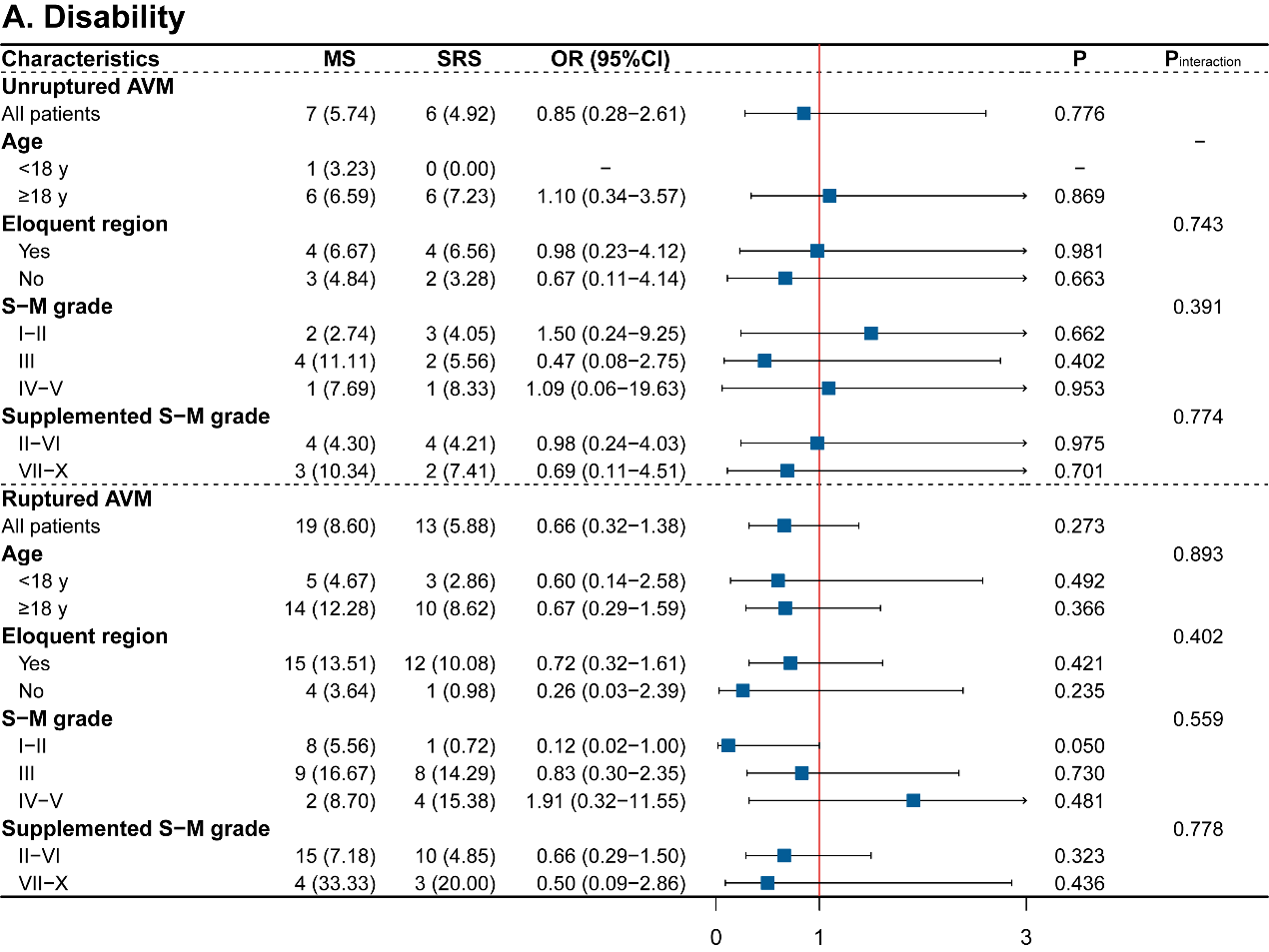


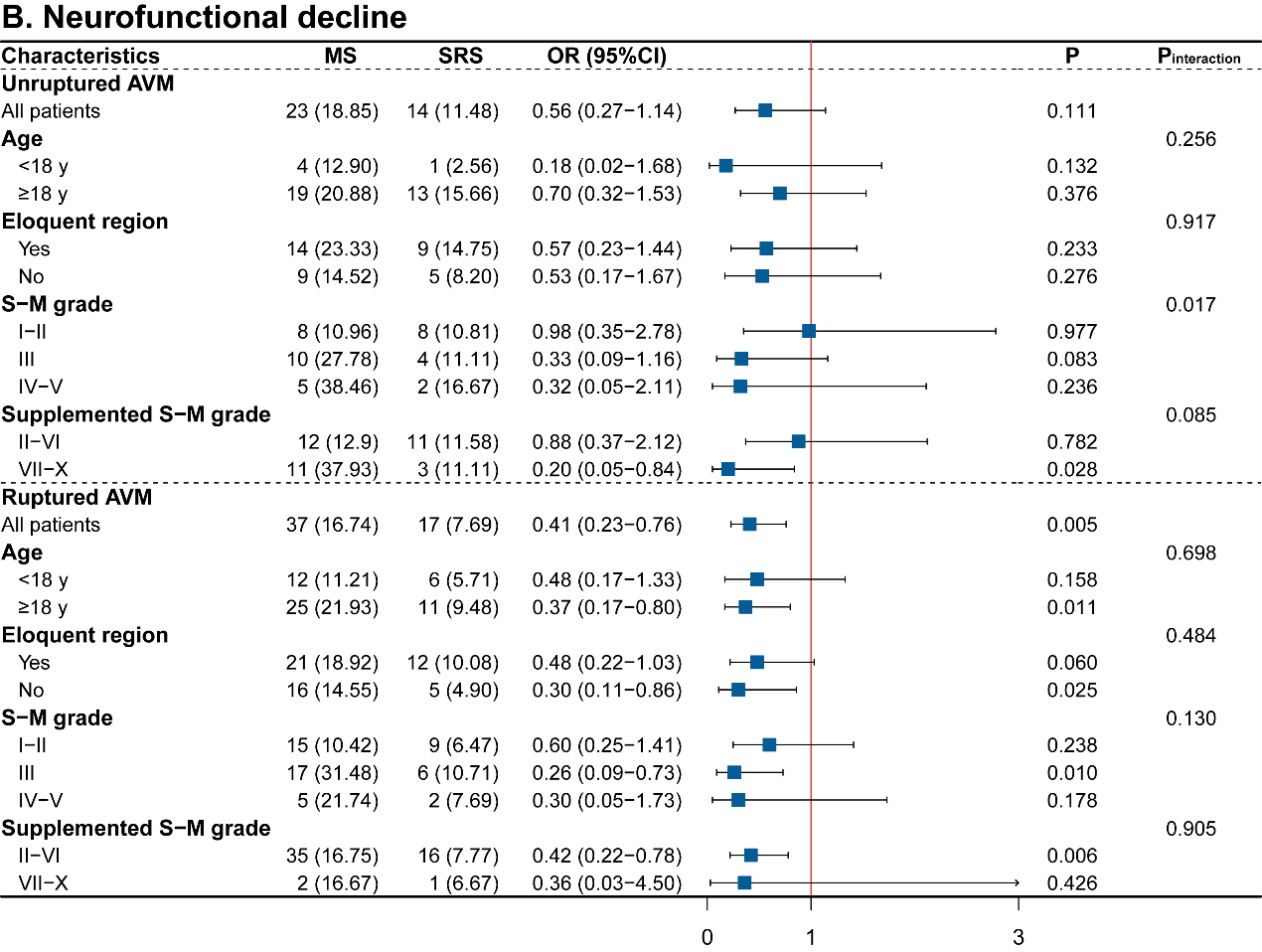


AVM, arteriovenous malformation; CI, confidence interval; mRS, modified Rankin Scale; MS, microsurgery; OR, odds ratio; S-M grade, Spetzler-Martin grade; SRS, stereotactic radiosurgery.

**Supplementary Table 6. Subgroup analyses for hemorrhagic stroke or death categorized by S-M grade.**

|  |  |  |  | Unruptured AVMs | | | | | | |  | Ruptured AVMs | | | | | | |
| --- | --- | --- | --- | --- | --- | --- | --- | --- | --- | --- | --- | --- | --- | --- | --- | --- | --- | --- |
|  |  |  |  |  | MS | | SRS | |  |  |  |  | MS | | SRS | |  |  |
| S-M grade | S | E | V | N | Event | PY | Event | PY | AR (95% CI) | P |  | N | Event | PY | Event | PY | AR (95% CI) | P |
| I | 1 | 0 | 0 | 59 | 0 | 155 | 3 | 204 | 1.47 (-0.19-3.13) | 0.083 |  | 85 | 1 | 260 | 2 | 231 | 0.48 (-0.94-1.90) | 0.506 |
| II | 1 | 1 | 0 | 36 | 0 | 124 | 3 | 154 | 1.94 (-0.26-4.14) | 0.083 |  | 96 | 0 | 318 | 4 | 307 | 1.30 (0.03-2.58) | 0.046 |
|  | 1 | 0 | 1 | 12 | 0 | 28 | 1 | 42 | 2.39 (-2.30-7.08) | 0.317 |  | 75 | 3 | 201 | 5 | 193 | 1.10 (-1.73-3.93) | 0.446 |
|  | 2 | 0 | 0 | 40 | 0 | 124 | 2 | 126 | 1.59 (-0.61-3.79) | 0.157 |  | 27 | 0 | 98 | 1 | 96 | 1.04 (-1.00-3.08) | 0.317 |
| III | 1 | 1 | 1 | 19 | 1 | 70 | 0 | 42 | -1.42 (-4.21-1.36) | 0.317 |  | 61 | 0 | 172 | 1 | 186 | 0.54 (-0.52-1.59) | 0.317 |
|  | 2 | 1 | 0 | 42 | 1 | 184 | 3 | 167 | 1.25 (-1.04-3.54) | 0.286 |  | 25 | 1 | 82 | 3 | 82 | 2.43 (-2.35-7.21) | 0.319 |
|  | 2 | 0 | 1 | 7 | 1 | 22 | 0 | 8 | -4.46 (-13.21-4.29) | 0.317 |  | 20 | 0 | 89 | 1 | 54 | 1.87 (-1.79-5.53) | 0.317 |
|  | 3 | 0 | 0 | 4 | 0 | 17 | 0 | 10 | - | - |  | 4 | 0 | 16 | 1 | 10 | 10.39 (-9.97-30.75) | 0.317 |
| IV | 2 | 1 | 1 | 14 | 0 | 45 | 1 | 75 | 1.34 (-1.29-3.97) | 0.317 |  | 38 | 0 | 99 | 5 | 119 | 4.19 (0.52-7.87) | 0.025 |
|  | 3 | 1 | 0 | 5 | 0 | 37 | 0 | 10 | - | - |  | 3 | 0 | 5 | 0 | 16 | - | - |
|  | 3 | 0 | 1 | 1 | 0 | 20 | 0 | 0 | - | - |  | 1 | 0 | 0 | 0 | 8 | - | - |
| V | 3 | 1 | 1 | 5 | 0 | 17 | 0 | 15 | - | - |  | 7 | 0 | 35 | 0 | 25 | - | - |

Abbreviations: AR, attributable risk; AVM, arteriovenous malformation; CI, confidence interval; MS, microsurgery; PY, patient-year; S-M grade, Spetzler-Martin grade (S, size; E, eloquence region; V, deep venous drainage); SRS, stereotactic radiosurgery.

**Supplementary Table 7. Subgroup analyses for disabling functional outcomes categorized by S-M grade.**

|  |  |  |  | Unruptured | | | | | | |  | Ruptured | | | | | | |  |
| --- | --- | --- | --- | --- | --- | --- | --- | --- | --- | --- | --- | --- | --- | --- | --- | --- | --- | --- | --- |
|  |  |  |  |  | MS | | SRS | |  |  |  |  | MS | | SRS | |  |  | |
| S-M grade | S | E | V | N | Event | Patient | Event | Patient | AR (95% CI) | P |  | N | Event | Patients | Event | Patient | AR (95% CI) | P |  |
| I | 1 | 0 | 0 | 59 | 0 | 30 | 0 | 29 | - | - |  | 85 | 2 | 45 | 1 | 40 | -1.94 (-9.78-5.89) | 0.623 |  |
| II | 1 | 1 | 0 | 36 | 0 | 18 | 1 | 18 | 5.56 (-5.42-16.53) | 0.311 |  | 96 | 4 | 49 | 0 | 47 | -8.16 (-15.93--0.40) | 0.040 |  |
|  | 1 | 0 | 1 | 12 | 1 | 5 | 0 | 7 | -20 (-59.86-19.86) | 0.290 |  | 75 | 1 | 34 | 0 | 41 | -2.94 (-8.72-2.83) | 0.313 |  |
|  | 2 | 0 | 0 | 40 | 1 | 20 | 2 | 20 | 5.00 (-11.79-21.79) | 0.550 |  | 27 | 1 | 16 | 0 | 11 | -6.25 (-18.71-6.21) | 0.312 |  |
| III | 1 | 1 | 1 | 19 | 2 | 10 | 0 | 9 | -20.00 (-46.69-6.69) | 0.132 |  | 61 | 8 | 29 | 5 | 32 | -11.96 (-32.96-9.03) | 0.259 |  |
|  | 2 | 1 | 0 | 42 | 1 | 20 | 2 | 22 | 4.09 (-11.73-19.92) | 0.604 |  | 25 | 1 | 10 | 3 | 15 | 10.00 (-19.01-39.01) | 0.483 |  |
|  | 2 | 0 | 1 | 7 | 1 | 4 | 0 | 3 | -25.00 (-80.65-30.65) | 0.300 |  | 20 | 0 | 12 | 0 | 8 | - | - |  |
|  | 3 | 0 | 0 | 4 | 0 | 2 | 0 | 2 | - | - |  | 4 | 0 | 3 | 0 | 1 | - | - |  |
| IV | 2 | 1 | 1 | 14 | 0 | 6 | 1 | 8 | 12.50 (-12.98-37.98) | 0.306 |  | 38 | 1 | 18 | 4 | 20 | 14.44 (-6.74-35.63) | 0.175 |  |
|  | 3 | 1 | 0 | 5 | 0 | 3 | 0 | 2 | - | - |  | 3 | 0 | 1 | 0 | 2 | - | - |  |
|  | 3 | 0 | 1 | 1 | 0 | 1 | 0 | 0 | - | - |  | 1 | 0 | 0 | 0 | 1 | - | - |  |
| V | 3 | 1 | 1 | 5 | 1 | 3 | 0 | 2 | -33.33 (-86.67-20.01) | 0.308 |  | 7 | 1 | 4 | 0 | 3 | -25.00 (-80.65-30.65) | 0.300 |  |

Abbreviations: AR, attributable risk; AVM, arteriovenous malformation; CI, confidence interval; MS, microsurgery; PY, patient year; S-M grade, Spetzler-Martin grade (S, size; E, eloquence region; V, deep venous drainage); SRS, stereotactic radiosurgery.

**Supplementary Table 8. Subgroup analyses for neurofunctional decline categorized by S-M grade and Supp S-M grade.**

|  |  |  |  | Unruptured | | | | | | |  | Ruptured | | | | | | |
| --- | --- | --- | --- | --- | --- | --- | --- | --- | --- | --- | --- | --- | --- | --- | --- | --- | --- | --- |
|  |  |  |  |  | MS | | SRS | |  |  |  |  | MS | | SRS | |  |  |
| S-M grade | S | E | V | N | Event | Patient | Event | Patient | AR (95% CI) | P |  | N | Event | Patients | Event | Patients | AR (95% CI) | P |
| I | 1 | 0 | 0 | 59 | 3 | 30 | 2 | 29 | -3.1 (-17.56-11.36) | 0.669 |  | 85 | 6 | 45 | 3 | 40 | -5.83 (-18.88-7.21) | 0.376 |
| II | 1 | 1 | 0 | 36 | 2 | 18 | 3 | 18 | 5.56 (-17.80-28.91) | 0.632 |  | 96 | 4 | 49 | 4 | 47 | 0.35 (-10.86-11.56) | 0.951 |
|  | 1 | 0 | 1 | 12 | 1 | 5 | 1 | 7 | -5.71 (-55.28-43.86) | 0.802 |  | 75 | 4 | 34 | 2 | 41 | -6.89 (-19.78-6.01) | 0.291 |
|  | 2 | 0 | 0 | 40 | 2 | 20 | 2 | 20 | 0.00 (-19.21-19.21) | >0.999 |  | 27 | 1 | 16 | 0 | 11 | -6.25 (-18.71-6.21) | 0.312 |
| III | 1 | 1 | 1 | 19 | 2 | 10 | 0 | 9 | -20.00 (-46.69-6.69) | 0.132 |  | 61 | 9 | 29 | 3 | 32 | -21.66 (-41.7--1.61) | 0.035 |
|  | 2 | 1 | 0 | 42 | 6 | 20 | 4 | 22 | -11.82 (-38.37-14.74) | 0.374 |  | 25 | 3 | 10 | 3 | 15 | -10.00 (-46.81-26.81) | 0.580 |
|  | 2 | 0 | 1 | 7 | 1 | 4 | 0 | 3 | -25.00 (-80.65-30.65) | 0.300 |  | 20 | 5 | 12 | 0 | 8 | -41.67 (-71.57--11.77) | 0.009 |
|  | 3 | 0 | 0 | 4 | 1 | 2 | 0 | 2 | -50.00 (-202.12-102.12) | 0.293 |  | 4 | 0 | 3 | 0 | 1 | - | - |
| IV | 2 | 1 | 1 | 14 | 1 | 6 | 2 | 8 | 8.33 (-38.69-55.36) | 0.706 |  | 38 | 5 | 18 | 2 | 20 | -17.78 (-43.15-7.59) | 0.164 |
|  | 3 | 1 | 0 | 5 | 1 | 3 | 0 | 2 | -33.33 (-119.95-53.28) | 0.308 |  | 3 | 0 | 1 | 0 | 2 | - | - |
|  | 3 | 0 | 1 | 1 | 1 | 1 | 0 | 0 | - | - |  | 1 | 0 | 0 | 0 | 1 | - | - |
| V | 3 | 1 | 1 | 5 | 2 | 3 | 0 | 2 | -66.67 (-153.28-19.95) | 0.092 |  | 7 | 0 | 4 | 0 | 3 | - | - |

Abbreviations: AR, attributable risk; AVM, arteriovenous malformation; CI, confidence interval; MS, microsurgery; S-M grade, Spetzler-Martin grade (S, size; E, eloquence region; V, deep venous drainage); SRS, stereotactic radiosurgery.

**Supplementary Table 9. Sensitivity analyses with different study designs.**

| **Sensitivity analysis** | **MS**  **Events (%)** | **SRS**  **Events (%)** | **AR (95%CI) *** | **P** | **HR (95%CI)/**  **OR (95%CI) *** | **P** |
| --- | --- | --- | --- | --- | --- | --- |
| **1.** **No treatment switching** | | | **MS vs SRS** | | | |
| **Hemorrhage stroke or death** | | |  |  |  |  |
| Unruptured | 2 (0.35) | 8 (1.31) | 0.96 (-0.07-1.99) | 0.069 | 3.59 (0.76-17.01) | 0.107 |
| Ruptured | 9 (0.69) | 22 (1.84) | 1.15 (0.27-2.04) | 0.011 | 2.84 (1.25-6.44) | 0.013 |
| Overall | 11 (0.59) | 30 (1.66) | 1.08 (0.39-1.77) | 0.002 | 3.04 (1.47-6.27) | 0.003 |
| **Obliteration** | | |  |  |  |  |
| Unruptured | 91 (98.91) | 30 (49.18) | -49.73 (-62.46--37.01) | <0.001 | 0.01 (0.00-0.08) | <0.001 |
| Ruptured | 191 (97.45) | 81 (50.94) | -46.51 (-54.58--38.43) | <0.001 | 0.03 (0.01-0.07) | <0.001 |
| Overall | 282 (97.92) | 111 (50.45) | -47.46 (-54.27--40.65) | <0.001 | 0.02 (0.01-0.05) | <0.001 |
| **Disability** | | |  |  |  |  |
| Unruptured | 5 (5.43) | 6 (6.52) | 1.09 (-7.94-5.76) | 0.756 | 1.21 (0.36-4.13) | 0.756 |
| Ruptured | 21 (10.71) | 12 (6.12) | -4.59 (-10.07-0.89) | 0.102 | 0.54 (0.26-1.14) | 0.106 |
| Overall | 26 (9.03) | 18 (6.25) | -2.78 (-7.11-1.55) | 0.210 | 0.67 (0.36-1.25) | 0.212 |
| **Neurofunctional decline** | | |  |  |  |  |
| Unruptured | 17 (18.48) | 11 (11.96) | -6.52 (-16.86-3.82) | 0.218 | 0.60 (0.26-1.36) | 0.221 |
| Ruptured | 38 (19.39) | 15 (7.65) | -11.73 (-18.4--5.07) | 0.001 | 0.34 (0.18-0.65) | 0.001 |
| Overall | 55 (19.1) | 26 (9.03) | -10.07 (-15.69--4.45) | 0.001 | 0.42 (0.26-0.69) | 0.001 |
| **2.** **Single session procedure** | | | **MS vs SRS** | | | |
| **Hemorrhage stroke or death** | | |  |  |  |  |
| Unruptured | 1 (0.22) | 5 (1.04) | 0.82 (-0.19-1.82) | 0.112 | 4.95 (0.58-42.39) | 0.144 |
| Ruptured | 1 (0.10) | 2 (0.23) | 0.13 (-0.24-0.50) | 0.499 | 2.12 (0.19-23.38) | 0.540 |
| Overall | 2 (0.14) | 7 (0.51) | 0.38 (-0.05-0.80) | 0.083 | 3.78 (0.78-18.20) | 0.097 |
| **Obliteration** | | |  |  |  |  |
| Unruptured | 79 (100.00) | 23 (43.4) | -56.6 (-69.95--43.26) | <0.001 | - | - |
| Ruptured | 158 (99.37) | 67 (54.03) | -45.34 (-54.2--36.48) | <0.001 | 0.01 (0.00-0.05) | <0.001 |
| Overall | 237 (99.58) | 90 (50.85) | -48.73 (-56.14--41.32) | <0.001 | 0.00 (0.00-0.03) | <0.001 |
| **Disability** | | |  |  |  |  |
| Unruptured | 6 (7.59) | 5 (6.33) | -1.27 (-9.20-6.67) | 0.755 | 0.82 (0.24-2.81) | 0.755 |
| Ruptured | 17 (10.69) | 5 (3.14) | -7.55 (-13.06--2.03) | 0.008 | 0.27 (0.10-0.75) | 0.012 |
| Overall | 23 (9.66) | 10 (4.2) | -5.46 (-10.00--0.92) | 0.019 | 0.41 (0.19-0.88) | 0.022 |
| **Neurofunctional decline** | | |  |  |  |  |
| Unruptured | 18 (22.78) | 8 (10.13) | -12.66 (-24.05--1.27) | 0.032 | 0.38 (0.16-0.94) | 0.036 |
| Ruptured | 26 (16.35) | 11 (6.92) | -9.43 (-16.41--2.46) | 0.009 | 0.38 (0.18-0.80) | 0.011 |
| Overall | 44 (18.49) | 19 (7.98) | -10.5 (-16.52--4.49) | 0.001 | 0.38 (0.22-0.68) | 0.001 |
| **3.** **Excluding SRS AVMs with follow-up < 3y** | | | **MS vs SRS** | | | |
| **Hemorrhage stroke or death** | | |  |  |  |  |
| Unruptured | 3 (0.41) | 14 (1.66) | 1.24 (0.26-2.23) | 0.014 | 3.46 (0.98-12.16) | 0.053 |
| Ruptured | 11 (1.01) | 20 (1.58) | 0.57 (-0.34-1.49) | 0.221 | 1.80 (0.81-4.00) | 0.150 |
| Overall | 14 (0.77) | 34 (1.61) | 0.84 (0.16-1.52) | 0.015 | 2.25 (1.15-4.40) | 0.018 |
| **Obliteration** | | |  |  |  |  |
| Unruptured | 104 (98.11) | 29 (36.25) | -61.86 (-72.71--51.02) | <0.001 | 0.01 (0.00-0.05) | <0.001 |
| Ruptured | 154 (91.67) | 73 (51.05) | -40.62 (-49.82--31.42) | <0.001 | 0.09 (0.05-0.18) | <0.001 |
| Overall | 258 (94.16) | 102 (45.74) | -48.42 (-55.52--41.32) | <0.001 | 0.05 (0.03-0.09) | <0.001 |
| **Disability** | | |  |  |  |  |
| Unruptured | 5 (4.72) | 5 (4.72) | 0.00 (-5.71-5.71) | >0.999 | 1.00 (0.28-3.56) | >0.999 |
| Ruptured | 18 (10.71) | 9 (5.36) | -5.36 (-11.14-0.43) | 0.071 | 0.47 (0.21-1.08) | 0.076 |
| Overall | 23 (8.39) | 14 (5.11) | -3.28 (-7.48-0.91) | 0.125 | 0.59 (0.3-1.17) | 0.129 |
| **Neurofunctional decline** | | |  |  |  |  |
| Unruptured | 23 (21.7) | 14 (13.21) | -8.49 (-18.65-1.66) | 0.103 | 0.55 (0.27-1.14) | 0.106 |
| Ruptured | 33 (19.64) | 13 (7.74) | -11.9 (-19.14--4.66) | 0.002 | 0.34 (0.17-0.68) | 0.002 |
| Overall | 56 (20.44) | 27 (9.85) | -10.58 (-16.52--4.65) | 0.001 | 0.43 (0.26-0.70) | 0.001 |
| **4.     sIPTW** |  |  | **MS vs SRS** | | | |
| **Hemorrhage stroke or death** | | |  |  |  |  |
| Unruptured | 13 (0.62) | 20 (1.39) | 0.77 (0.07-1.48) | 0.031 | 2.30 (0.61-8.70) | 0.221 |
| Ruptured | 17 (0.53) | 43 (1.98) | 1.45 (0.80-2.09) | <0.001 | 3.54 (1.58-7.94) | 0.002 |
| Overall | 28 (0.53) | 66 (1.90) | 1.37 (0.87-1.87) | <0.001 | 3.48 (1.75-6.90) | <0.001 |
| **Obliteration** | | |  |  |  |  |
| Unruptured | 305 (96.57) | 46 (28.97) | -67.61 (-74.92--60.30) | <0.001 | 0.01 (0.01-0.03) | <0.001 |
| Ruptured | 486 (91.8) | 113 (42.36) | -49.44 (-55.82--43.06) | <0.001 | 0.07 (0.04-0.10) | <0.001 |
| Overall | 796 (93.66) | 149 (35.88) | -57.78 (-62.69--52.88) | <0.001 | 0.04 (0.03-0.05) | <0.001 |
| **Disability** | | |  |  |  |  |
| Unruptured | 19 (6.14) | 10 (4.55) | -1.59 (-5.46-2.28) | 0.434 | 0.73 (0.33-1.61) | 0.431 |
| Ruptured | 75 (14.19) | 23 (6.70) | -7.49 (-11.48--3.50) | 0.001 | 0.43 (0.27-0.71) | 0.001 |
| Overall | 104 (12.25) | 34 (6.40) | -5.86 (-8.89--2.83) | <0.001 | 0.49 (0.33-0.73) | 0.001 |
| **Neurofunctional decline** | | |  |  |  |  |
| Unruptured | 53 (16.64) | 19 (9.23) | -7.41 (-13.09--1.73) | 0.016 | 0.51 (0.29-0.89) | 0.018 |
| Ruptured | 127 (24.05) | 25 (7.26) | -16.79 (-21.36--12.22) | <0.001 | 0.25 (0.16-0.39) | <0.001 |
| Overall | 189 (22.24) | 48 (9.05) | -13.19 (-16.9--9.48) | <0.001 | 0.35 (0.25-0.49) | <0.001 |

^*^ The results were calculated with the MS group as the reference. The metrics of the primary outcomes were expressed as rate per 100 patient-years and hazard ratios, and the secondary outcomes were expressed as proportion and odds ratios.

Abbreviation: AVM, arteriovenous malformation; CI, confidence interval; HR, hazard ratio; MS, microsurgery; OR, odds ratios; sIPTW, stabilized inverse probability of treatment weighting; SRS, stereotactic radiosurgery.
